# Affected pathways and transcriptional regulators in gene expression response to an ultra-marathon trail: Global and independent activity approaches

**DOI:** 10.1371/journal.pone.0180322

**Published:** 2017-10-13

**Authors:** Maria Maqueda, Emma Roca, Daniel Brotons, Jose Manuel Soria, Alexandre Perera

**Affiliations:** 1 Department of ESAII, Center for Biomedical Engineering Research, Universitat Politècnica de Catalunya, Barcelona, Catalonia, Spain; 2 CIBER de Bioingeniería, Biomateriales y Nanomedicina (CIBER-BBN), Barcelona, Catalonia, Spain; 3 Summit 2014 S.L., Centelles, Barcelona, Catalonia Spain; 4 Department of Electronic Engineering, Center for Biomedical Engineering Research, Universitat Politècnica de Catalunya, Barcelona, Catalonia, Spain; 5 Catalan Sports Council, Barcelona, Catalonia, Spain; 6 Unit of Genomics of Complex Diseases, Institut de Recerca de l'Hospital de la Santa Creu i Sant Pau, Barcelona, Catalonia, Spain; College of Bioinformatics Science and Technology, CHINA

## Abstract

Gene expression (GE) analyses on blood samples from marathon and half-marathon runners have reported significant impacts on the immune and inflammatory systems. An ultra-marathon trail (UMT) represents a greater effort due to its more testing conditions. For the first time, we report the genome-wide GE profiling in a group of 16 runners participating in an 82 km UMT competition. We quantified their differential GE profile before and after the race using HuGene2.0st microarrays (Affymetrix Inc., California, US). The results obtained were decomposed by means of an independent component analysis (ICA) targeting independent expression modes. We observed significant differences in the expression levels of 5,084 protein coding genes resulting in an overrepresentation of 14% of the human biological pathways from the Kyoto Encyclopedia of Genes and Genomes database. These were mainly clustered on terms related with protein synthesis repression, altered immune system and infectious diseases related mechanisms. In a second analysis, 27 out of the 196 transcriptional regulators (TRs) included in the Open Regulatory Annotation database were overrepresented. Among these TRs, we identified transcription factors from the hypoxia-inducible factors (HIF) family EPAS1 (p< 0.01) and HIF1A (p<0.001), and others jointly described in the gluconeogenesis program such as HNF4 (p< 0.001), EGR1 (p<0.001), CEBPA (p< 0.001) and a highly specific TR, YY1 (p<0.01). The five independent components, obtained from ICA, further revealed a down-regulation of 10 genes distributed in the complex I, III and V from the electron transport chain. This mitochondrial activity reduction is compatible with HIF-1 system activation. The vascular endothelial growth factor (VEGF) pathway, known to be regulated by HIF, also emerged (p<0.05). Additionally, and related to the brain rewarding circuit, the endocannabinoid signalling pathway was overrepresented (p<0.05).

## Introduction

Previous research has identified mechanisms triggered with the practice of moderate exercise that yield beneficial effects on health, specially on cardiovascular disease [1]. These effects may be explained due to the adaptation of many organs to cope with required musculoskeletal performance [2]. However, health benefits for the case of extreme endurance exercise remain unclear [3,4].

Ultra-marathon trails (UMTs) could be considered as extreme endurance exercise since their running events should be longer than the traditional marathon (42.195 km). Typically, they are run through a mountainous terrain with a considerable accumulated altitude change. Due to their high physical and psychological demand, UMTs are identified as an ideal sport for investigating a wide range of physiological responses [5]. These competitions show a growing popularity as indicated by the, approximately, sevenfold increase in the number of finishers of 100km worldwide ultra-marathons between 1998 and 2011 [6]. In parallel, the amount of scientific contributions focusing on UMT interventions has risen. They cover a varied range of perspectives: some authors detected reactive oxygen species (ROS) promotion, oxidative stress and inflammation in runners (n = 46) participating in a 330km UMT from capillary blood sample using micro-invasive analytic methods [7]; others evidenced a respiratory muscle strength reduction in inspiratory muscles when running a 110km UMT (n = 22) [8] or even, the adversely impact in the cognitive performance after a 168km UMT race (n = 17) [9].

To the best of our knowledge, there are no studies that approach UMT runners’ genome-wide gene expression (GE) response. This methodology has been used in other type of exercise-related interventions such as a single bout of 4-hour stationary cycling (n = 5) [10] or after a specific running endurance training (n = 13) [11]. On the other hand, GE on particular sets of genes has been assessed in shorter distances as marathon races when studying the response of specific interleukins (n = 16) [12] or in toll-like receptors (n = 47) [13].

A better understanding of the immune and inflammatory response has been the main motivation with regard to peripheral blood sample experiments. The link between exercise and immune system has long been studied tracing the beginning back to 1893 when an exercise-induced leukocytosis was described [14]. Prior studies suggest that moderate exercise negatively correlates with upper respiratory tract infections (URTI) incidence among other positive clinical implications [15]. However, this may not be the case in marathon or similar events where the opposite effect is detected [16]. A mechanistic explanation of an increased URTI risk in marathon runners (n = 16) is proposed elsewhere [17]. This study is based on the ratio imbalance of GE values from genes related to T-helper 1 (Th1) and Th2 cells. Likewise, other authors summarized the exercise impact on the GE of common inflammatory markers in a diverse range of exercise disciplines, intensity and duration [18].

In other experiments, the use of skeletal muscle biopsy samples is driven by the understanding of the adaptation of the human skeletal muscle to exercise [19]. In this context, the role of the hypoxia-inducible factor family (HIF), as reviewed in [20], is of great interest since its target genes include the vascular endothelial growth factor (VEGF), which related signalling pathway is one of the events driving the vascular system remodelling known to occur with dynamic exercise [2].

In this study, we obtained the genome-wide GE profiling in a group of runners (n = 16) participating in an 82km UMT race. We report the biological pathways and transcriptional regulators enriched by the list of differentially expressed genes as a result of the UMT intervention. For doing so, we addressed the genetic response from a global perspective and from an independent activity approach after implementing a statistical method capable of extracting independent sources of information.

## Materials and methods

### Ethical approval

All procedures involved in this study conformed to the Declaration of Helsinki. Ethical approval was granted by the Ethics Committee of the Catalan Sports Council from Government of Catalonia (Approval number 0099S/2046/2013). Written informed consent was obtained from all individuals participating in the study.

### Experimental design

We approached and recruited 18 healthy runners who accepted to voluntarily participate in the study. They were athletes with prior experience in UMTs and presented no muscle injuries in the previous six months. One of the subjects dropout so finally, a total of 17 runners participated in the study (12 males aged 38.2 ± 4.3 years, 5 females aged 35.6 ± 2.2 years). All individuals were of Western European descent. Table 1 shows basic anthropometrical measures and weekly training hours per participant. The experiment was conducted in June 2012 at the “Cavalls del Vent” UMT located in the Catalan Pyrenees (Spain). This was a circular 82 km route starting at 760m above sea level and achieving a maximum altitude of 2,520m. The total accumulated altitude change was 12,180 m. In previous edition (2011), male and female winners needed 8.9 hours and 11.6 hours respectively to complete the race while last finishers took approximately 22 hours (no gender differentiation) to cover the total distance.

**Table 1:** Participants in the study–age, basic anthropometric and training regime.

Participant id	Gender	Age [years]	Height [cm]	Weight [kg]	Training regime [hours per week]
1	Female	35.9	167	58.0	-
2	Female	38.4	164	60.0	15
3	Male	45	171	72.0	6
4	Male	41.8	172	70.0	7.5
5	Male	37.1	184	74.2	5.5
6	Male	40.3	164	55.0	15
7	Male	36.8	183	75.6	10
8	Female	39.1	173	67.0	15
9	Male	37.1	176	68.0	-
10	Male	41.5	182	91.4	5
11	Male	38.1	178	78.0	7
12	Male	40.3	179	71.0	5
13	Male	27.6	167	59.2	7.5
14	Male	35.8	180	84.0	5
15	Female	41.9	-	-	-
16	Male	37	165	53.7	-
17	Female	37.6	-	-	5

### Blood samples, RNA extraction and microarray expression data

Venous blood samples were drawn, at rest in a sitting position, from the antecubital vein and collected into PAXgene Blood RNA Tubes according to the manufacturer’s protocol (PreAnalytiX GmbH/QIAGEN, Switzerland/US). Samples were obtained from each subject prior to and immediately after the UMT, with the exception of five participants (ids 1, 9, 13, 15 and 16 as shown in Table 2) from whom only pre-race samples were available. A total of 29 samples, 17 of them corresponding to pre-race and 12 to post-race, were stored at -80°C until assayed in the Hospital de la Santa Creu i Sant Pau (Barcelona, Spain). Samples were tagged with an identifier followed by PRE or POST referring to pre- or post-race sample. Total RNA was isolated using the PAXgene Blood RNA kit (PreAnalytiX GmbH/QIAGEN, Switzerland/US). The concentration of the extracted RNA was measured spectrophotometrically (Nanodrop 1000/ Thermo Fisher Scientific, Wilmington, US). GeneChip WT Plus Reagent kit (Affymetrix Inc., California US) was used for processing 100ng of total RNA per sample. Biotinylated sscDNA was hybridized for 16 hour at 45°C and 60 rpm on HuGene2.0st microarrays in a Hybridization Oven 640, both from Affymetrix. Microarrays were washed and stained in the Affymetrix Fluidics Station 450. Finally, they were confocal scanned using the GeneChip 3000 7G with Autoloader from Affymetrix. Raw fluorescence intensity values were stored in Chip Expression Level (CEL) file types, one per available blood sample. Data is available in the Gene Expression Omnibus database (GSE93945).

**Table 2:** Completed distance, time achieved and average speed per study participant. Table indicates the race performance for each participant in the study. Biological sample availability is indicated in terms of pre- or post- race extraction.

Participant	Sample availability	Completed distance [km]	Time achieved [hours]	Average speed [km/h]
1	Pre-race	82	10.6	7.7
2	Pre- and Post-race	82	11.5	7.1
3	Pre- and Post-race	50	10.0	5
4	Pre- and Post-race	50	10.0	5
5	Pre- and Post-race	25	7.0	3.6
6	Pre- and Post-race	42	4.5	9.3
7	Pre- and Post-race	50	10.0	5
8	Pre- and Post-race	82	11.5	7.1
9^(1)^	Pre-race	58	5.3	10.9
10	Pre- and Post-race	25	7.0	3.6
11	Pre- and Post-race	33	5.0	6.6
12	Pre- and Post-race	50	10.0	5
13	Pre-race	28	6.1	4.6
14	Pre- and Post-race	50	10.0	5
15	Pre-race	Unknown	Unknown	Unknown
16	Pre-race	Unknown	Unknown	Unknown
17	Pre- and Post-race	14	3.1	4.5

^(1)^This sample was removed for downstream analysis due to negative Quality Control results.

The corresponding expression profiles from the CEL files were background corrected, quantile normalized and summarized using the Robust Multichip Average (RMA) [21] on the R software platform [22] with BioConductor [23] using the oligo package [24]. The expression levels of 53,617 transcript clusters (TCs) were available per sample. Quality control (QC) was performed over pre-processed data to detect possible outliers based on the following metrics: relative log expression [25], normalized unscaled standard error [25], density intensity distributions (histogram and boxplot) and principal component analysis (PCA). Relevant versions of used packages are given in S1 Table.

### Differential gene expression analysis (DGEA)

Only those TCs targeting protein-coding RNA molecules were considered for DGEA based on the annotation from the *hugene20sttranscriptcluster*.*db* package [26]. A non-supervised filtering [27] was applied to discard low expressed TCs which were assumed to be non-informative. TCs with expression values higher than the overall intensity mean, computed across all arrays, and on more than 12 arrays were selected for DGEA. The genefilter package [28] was used for this purpose. Then, a linear regression model (LM) was fitted to each TC expression value according to Eq (1).

$$g_{k}=\beta_{0k}+\beta_{1k}∙g+\beta_{2k}∙d+\epsilon_{k}$$ (1)

where *g*_*k*_ is the expression value of TC *k*, *β*_0*k*_ is the LM intercept for TC expression value *k*, *β*_1*k*_ and *β*_2*k*_ are the unknown coefficients for the variables gender *g* and distance *d* respectively and *ϵ*_*k*_ are the random errors. The empirical Bayes moderated t-statistics tested whether each individual coefficient was zero using the *limma* package [29]. Statistically significant differentially expressed TCs (differential TCs) were selected and ranked (adjusted p-value < 5%, FDR) per LM predictor variable. Entrez Gene identifiers (IDs) were mapped from their differential TCs.

**Fig 1:**
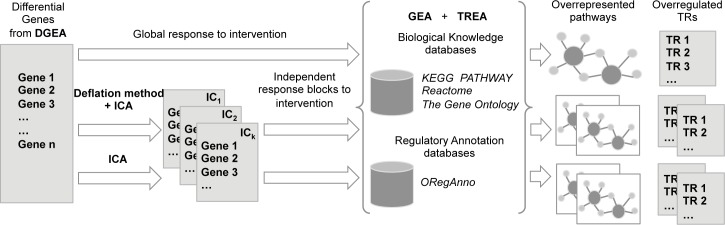
Complete workflow implemented for the study. The differential genes list obtained from the differential gene expression analysis is taken as the initial step for the workflow. This represents the global response to intervention but can also be decomposed in independent components through an Independent Component Analysis (ICA) to obtain the independent block response. ICA is computed after applying a deflation method to the original expression data. Gene and transcriptional regulator (TR) enrichment analyses are computed over the global and independent response. Results are summarized in overrepresented pathway graphs and overrepresented TRs rankings.

The resulting list of differential genes was used as input for the downstream analysis (Fig 1). A heatmap was generated with *gplots* package [30] for selected TCs including a hierarchical clustering with complete linkage method.

### Independent activity analysis

Microarray expression data could be understood as a linear combination of independent expression sources, each one associated with a particular biological reading [31]. We computed an Independent Component Analysis (ICA) to extract these expression sources [32] according to Eq (2).

$$X^{T}=SA$$ (2)

where **X** is an *n* × *m* matrix of the expression values of *n* genes under *m* array samples. The columns of the *m* × *k* source matrix **S** contain *k* independent components (ICs) and the *k* × *n* matrix **A** represents the linear mixing matrix. The row of matrix **A** comprises the weights with which the expression levels of the *n* genes contribute to each *k*^*th*^ expression mode.

The list of differential genes was selected to build a matrix **X**. First, the optimum number of *k* ICs for **X** was obtained by estimating the optimal number of components in the PCA using the generalized cross-validation approximation (GCV) and the smoothing method [33], both implemented in the *FactoMineR* package [34]. Then a deflationary method was applied to **X** to remove the first component of variance as computed by the PCA. This was applied to eliminate the main response to the intervention characterized by the immune system and the genetic information processes as latter shown. These powerful signals act as a masking effect for the rest of underlying processes making difficult for ICA to detect them. Deflation was applied according to Eq (3):

$$Y^{T}=z_{i1}⊗\phi_{j1}$$ (3)

where **Y** is an *n* × *m* matrix which refers to the expression values of *n* genes from *m* array samples captured by the first principal component (PC1), **z**_**i1**_ is the scores vector of the *i*_*th*_ array sample in PC1 and **ϕ**_**j1**_ corresponds to the loadings vector of the *j*_*th*_ gene. Lastly, an estimated matrix ${\hat{X}}^{T}$ was built according to Eq (4):

$${\hat{X}}^{T}=X^{T}-Y^{T}$$ (4)

ICA was performed over both the matrixes **X**^**T**^ and ${\hat{X}}^{T}$ where *k* − 1 ICs were considered in the second case due to the applied deflation. The *fastICA* package was used [35] (ε_t_ <1e-4, *G* ≈ *log cosh* with *α*_1_ = 1 [32], ICs extracted simultaneously). Those genes with absolute weight value included in the ninth decile were considered as the most representative genes for each specific IC.

### Gene enrichment analysis (GEA)

A GEA was applied in two stages: (i) globally when considering the list of differential genes and (ii) specifically for each IC derived from ICA and only considering the most representative genes. GEA was computed over Kyoto Encyclopedia of Genes and Genoms (KEGG) PATHWAY [36], Reactome [37] and The Gene Ontology (GO) Biological Processes [38] databases with the package *clusterProfiler* [39]. For each queried biological pathway or GO term, an adjusted p-value was calculated with a hypergeometric distribution test (adjusted p-value < 5%, False Discovery Rate FDR). The background distribution was defined by all available annotations in the relevant database or by the list of differential genes if the global or ICA stage was considered, respectively.

### Transcriptional regulator enrichment analysis (TREA)

To explore overrepresented transcriptional regulators (TRs) as a response to UMT completed distance, a TREA was conducted. We considered differential genes to be potentially regulated by one or more TRs. The TREA was implemented with a hypergeometric model to assess whether the number of differential genes related to a specific TR was larger than expected. TRs were ranked based on their adjusted p-values (<5%, FDR). The TREA was implemented in two stages, globally and specifically per IC. A compilation of interactions between human TRs and target genes (TGs) was obtained from the Open Regulatory Annotation (ORA) database v3.0 [40]. Interactions between 196 regulatory elements and 23,991 TGs were chosen (*type* of regulation was set to *transcription factor binding site*, GRCh37/hg19). Background distribution was defined by the complete customized database or by the list of differential genes if the global or ICA stage was considered, respectively.

## Results

Only 3 out of the 17 initial participants in the study finished the UMT while the rest of volunteers decided to leave the race at different distances along the trail. This was due to the adverse weather conditions mainly because of low temperatures (from 0.9°C to 13.1°C) and rain presence (from 0 to 6.1mm/h). The corresponding completed distance per participant and respective achieved time is given in Table 2. Additionally, biological sample availability is commented relative to its extraction before and/or after the race (pre- and post-race respectively).

**Fig 2:**
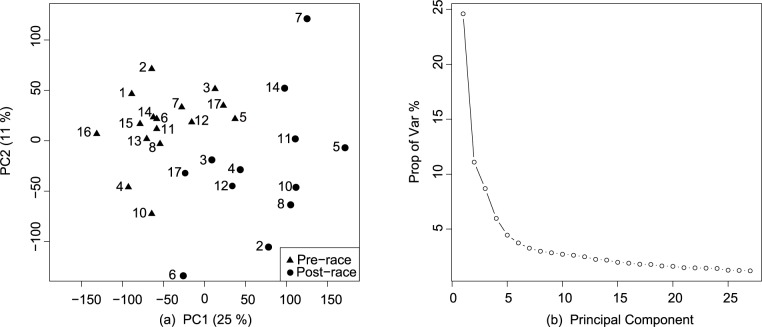
Principal component analysis (PCA) over the expression values of the ranked transcript clusters (TCs). (a) PCA over 25,272 TCs expression values targeting protein coding RNA molecules included in HuGene20st microarray. First principal component was aligned with the effect of participating in the Ultra Marathon Trail (UMT) (b) Proportion (in %) of captured variance per principal component.

QC excluded one pre-race sample, without post-race counterpart, which showed an abnormal pattern (S1 Fig). Pre-processing and QC was repeated after its removal with positive results. Therefore, a total of 28 samples, 16 of them pre-race and 12 post-race, were kept for further analysis. After filtering by target protein-coding RNA molecules, 25,272 TCs were available for DGEA. This group of TCs interrogated 22,072 different genes. To visualize their main source of variance, a PCA was conducted over their expression values. PC1 captured 25% of the total data variance, this being aligned with the effect of participating in the UMT (Fig 2).

### DGEA reveals a list of 5084 distinct genes responding to intervention

**Fig 3:**
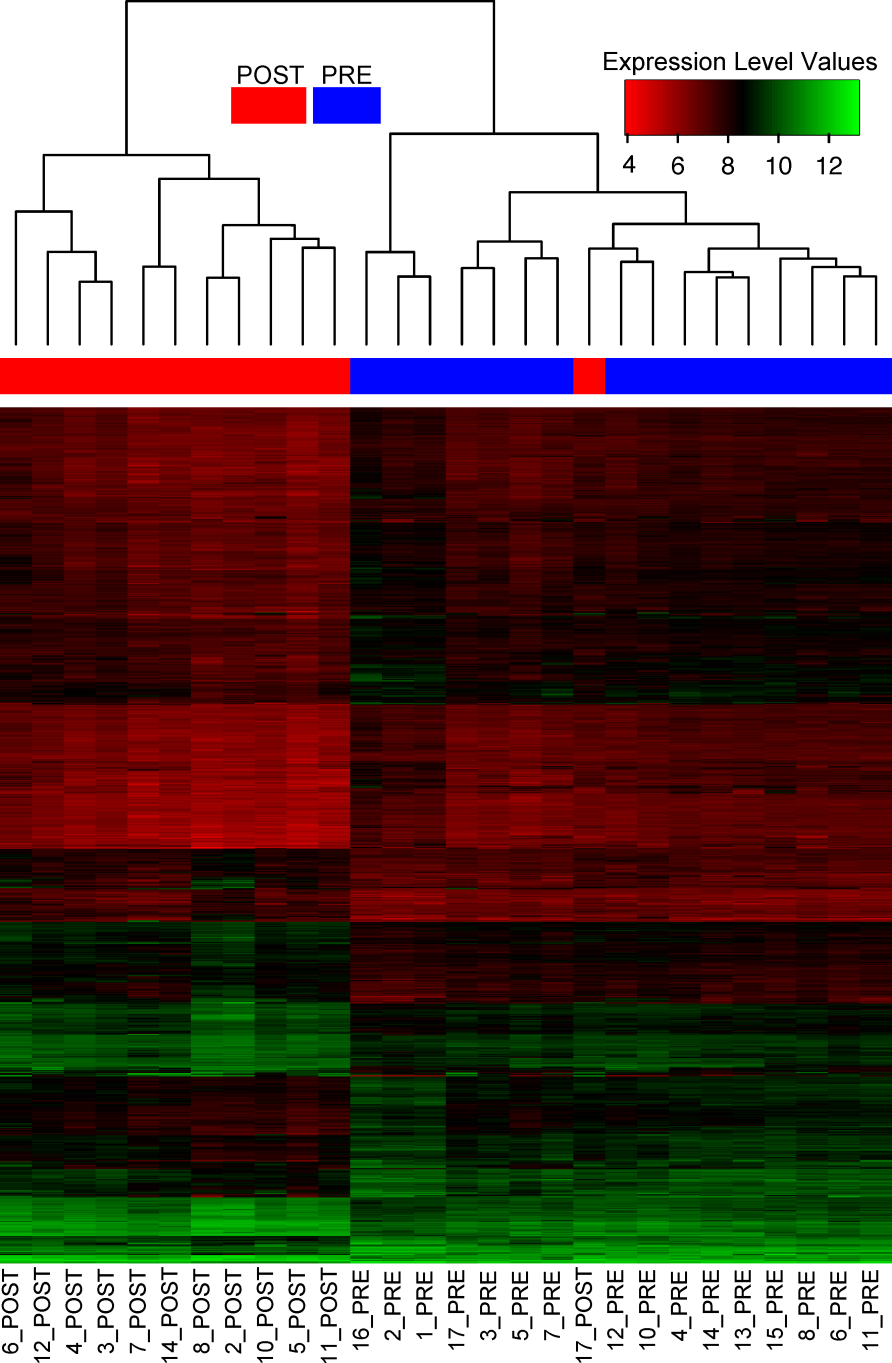
Heatmap of the ranked transcript clusters (TCs) with stronger effect as response to the intervention. Heatmap of the TCs with stronger effect as response to completed distance in the Ultra Marathon Trail (UMT). Selection was based on β_2_ values obtained in the linear model. Those TCs with *abs*(β_2_) > $\mu_{\beta_{2}}+{2\sigma }_{\beta_{2}}$ were chosen corresponding to 1,115 among the 5,499 differential TCs.

DGEA identified 5,974 differential TCs as a response to UMT (β_2_ ≠ 0). Among the list of 5,974 differential TCs, 5,499 were unambiguously annotated to a single gene (S2 Table) while 475 had multiple annotations (S3 Table). The list of 5,499 differential TCs corresponded to 5,084 distinct genes which were mainly down-regulated (63%) rather than up-regulated (37%). No TC appeared with statistical correlation with runners’ age. Fig 3 shows an unsupervised clustering analysis of a subset from the latter 5,499 differential TCs. The figure indicates a coherent sorting of samples prior to UMT compared to posterior ones, except for one misclassified sample labelled as *17_POST* which corresponds to an individual who only completed 17% of the UMT. DGEA additionally revealed 35 differential TCs (S4 Table) related to gender (β_1_ ≠ 0, all TCs with single gene annotation). A 43% of these differential genes showed a higher expression level in male than in female runners.

### Global response

#### Pathways associated with genetic information processing, infectious diseases and immune system are mainly affected

**Fig 4:**
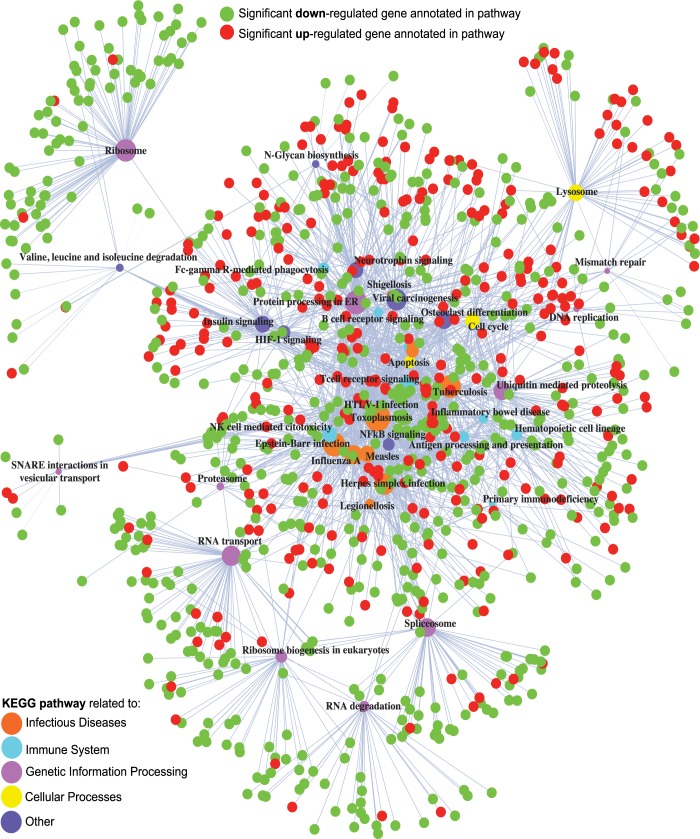
Network of the overrepresented Kyoto Encyclopedia of Genes and Genomes (KEGG) pathways listed in Table 3 connected through their differential annotated genes. Statistically significant overrepresented KEGG pathways within the 5,084 distinct genes with marked differential expression level as a response to endurance exercise. Pathway’s circle size is proportional to the number of annotated genes (node degree). Pathway’s node color refers to their specific main category according to the KEGG structure. Genes annotated to each pathway are color-coded according to their type of regulation (green codes for down-regulation and red for up-regulation).

The 5,804 differential genes were used to conduct a global GEA for each of the three databases: KEGG, Reactome and GO Biological Processes. Results obtained from KEGG revealed a list of 42 statistically overrepresented pathways (Table 3). All of them were connected through 978 out of the 5,084 initial differential genes (Fig 4). According to the database structure, 11 among the 42 induced pathways were involved in genetic information processing with most of their annotated genes down-regulated (mean 86.7% ± standard deviation 11.1%). A total of 11 affected infectious diseases were distributed among bacterial (three), viral (five) and parasitic (three) infection types. Genes annotated to bacterial and parasitic pathways were up-regulated by 61.5% ± 3.6% and 61.2% ± 8%, respectively. Genes annotated to the viral pathways were mostly down-regulated by 59.7% ± 5.3%. Nine pathways from the immune system emerged, including specific signalling pathways (three) and related immune diseases (two). No significant common sense of regulation was observed in this case with the exception of the two immune diseases, both mainly down-regulated (62.1% and 88.9%). Both lymphoid and myeloid cell lines from the *hematopoietic cell lineage* pathway were impacted (S2 Fig). Cell surface molecules included in this pathway (26 out of 55) were showing up-regulation (CD1, CD11b, CD13, CD14, CD35, CD36, CD42, CD55, CD59, CD114, CD116, CD121, CD124 and CD126) or down-regulation (CD2, CD3, CD5, CD7, CD8, CD20, CD24, CD38, CD49, CD71, CD125 and CD127). Other overrepresented pathways refer to signal transduction such as signalling pathways for HIF-1 and Nuclear Factor NF-κβ, with 58.1% and 53.8% of the annotated genes up-regulated respectively. Several cellular processes, as *apoptosis* (with 52.6% of annotated genes up-regulated) and *cell cycle* (with 79.6% of annotated genes down-regulated) were also impacted. The complete list of up-and down-regulated genes per listed pathway is enclosed in S5 Table.

**Table 3:** List of the 42 overrepresented Kyoto Encyclopedia of Genes and Genomes (KEGG) pathways as a response to the intervention. *KEGG pathway identifier (ID)* and *description* is enclosed in the table. Pathway’s *main category* and *subcategory* are shown. *Gene*:*Bg Ratio* indicates the number of genes annotated to a pathway (within the specific list of 1,905 out of 5,084 differential genes which appear in KEGG database) versus the number of genes annotated to that specific pathway within the background. Background considers all genes included in the database which corresponds to 6,997 elements. Pathways are sorted based on their *adj p-val* (FDR correction) coded as *** < 0.001, ** < 0.01 and * < 0.05. *Up-reg[%]* indicates the percentage of differential genes annotated to the specific pathway being up-regulated.

KEGG Pathway ID:Description	Main Category—Subcategory	Gene: Bg Ratio	adj p-val	Up-reg [%]
hsa03010:Ribosome	GIP—Translation	75:137	***	4
hsa04141:Protein processing in ER	GIP—Folding, sorting and degradation	80:169	***	26.2
hsa04660:T cell receptor SP	OS—Immune system	52:104	***	32.7
hsa03040:Spliceosome	GIP—Transcription	61:134	***	14.8
hsa05152:Tuberculosis	HD—Infectious Diseases: Bacterial	76:178	***	60.5
hsa04380:Osteoclast differentiation	OS—Development	59:131	***	72.9
hsa04621:NOD-like receptor SP	OS—Immune system	30:57	**	63.3
hsa04120:Ubiquitin mediated proteolysis	GIP—Folding, sorting and degradation	59:137	**	22
hsa04142:Lysosome	CP—Transport and catabolism	54:123	**	64.8
hsa05134:Legionellosis	HD—Infectious Diseases: Bacterial	29:55	**	65.5
hsa05169:Epstein-Barr virus infection	HD—Infectious Diseases: Viral	80:202	**	40
hsa05145:Toxoplasmosis	HD—Infectious Diseases: Parasitic	51:118	**	52.9
hsa03430:Mismatch repair	GIP—Replication and repair	15:23	**	0
hsa04722:Neurotrophin SP	OS—Nervous system	51:120	**	60.8
hsa05168:Herpes simplex infection	HD—Infectious Diseases: Viral	72:185	**	36.1
hsa04210:Apoptosis	CP—Cell growth and death	38:85	**	52.6
hsa03013:RNA transport	GIP—Translation	67:172	**	14.9
hsa04066:HIF-1 SP	EIP—Signal transduction	43:103	*	58.1
hsa04910:Insulin SP	OS—Endocrine system	55:139	*	56.4
hsa05166:HTLV-I infection	HD—Infectious Diseases: Viral	93:258	*	36.6
hsa03018:RNA degradation	GIP—Folding, sorting and degradation	34:77	*	11.8
hsa03050:Proteasome	GIP—Folding, sorting and degradation	22:44	*	9.1
hsa03008:Ribosome biogenesis in eukaryotes	GIP—Translation	38:89	*	7.9
hsa04064:NF-kappa B SP	EIP—Signal transduction	39:93	*	53.8
hsa05140:Leishmaniasis	HD—Infectious Diseases: Parasitic	32:73	*	68.8
hsa00280:Valine, leucine and isoleucine degradation	M—Amino acid metabolism	23:48	*	21.7
hsa05162:Measles	HD—Infectious Diseases: Viral	53:136	*	39.6
hsa04110:Cell cycle	CP—Cell growth and death	49:124	*	20.4
hsa05131:Shigellosis	HD—Infectious Diseases: Bacterial	29:65	*	58.6
hsa05321:Inflammatory bowel disease (IBD)	HD—Immune diseases	29:65	*	37.9
hsa04612:Antigen processing and presentation	OS—Immune System	33:77	*	12.1
hsa05142:Chagas disease	HD—Infectious Diseases: Parasitic	42:104	*	61.9
hsa04650:Natural killer cell mediated cytotoxicity	OS—Immune System	52:135	*	36.5
hsa04666:Fc gamma R-mediated phagocytosis	OS—Immune System	38:93	*	63.2
hsa03030:DNA replication	GIP—Replication and repair	18:36	*	0
hsa04640:Hematopoietic cell lineage	OS—Immune System	36:88	*	50
hsa04130:SNARE interactions in vesicular transport	GIP—Folding, sorting and degradation	17:34	*	35.3
hsa05340:Primary immunodeficiency	HD—Immune diseases	18:37	*	11.1
hsa00510:N-Glycan biosynthesis	M—Glycan biosynthesis and metabolism	22:49	*	9.1
hsa05164:Influenza A	HD—Infectious Diseases: Viral	63:175	*	49.2
hsa04662:B cell receptor SP	OS—Immune system	30:73	*	56.7
hsa05203:Viral carcinogenesis	HD—Cancers: Overview	72:205	*	52.8

Abbreviations: GIP, Genetic Information Processing; OS, Organismal System; HD, Human Diseases; CP, Cellular Processes; EIP, Environmental Information Processing; M, Metabolism; SP, Signaling Pathway.

A total of 193 Reactome pathways were found statistically overrepresented (S6 Table). Table 4 shows a summary by clustering them into parental superclasses based on the database hierarchy. *Gene Expression*, *Immune System* and *Disease* were top affected superclasses which enclose biological information similar to abovementioned KEGG genetic information processing, immune system and infectious disease. Obtained Reactome pathways related to *Disease* were all concentrated on viral infectious diseases capturing 21 out of the 193 ranked pathways.

**Table 4:** Clustering of the 193 statistically overrepresented Reactome pathways into parental superclasses. Table shows the number of overrepresented pathways annotated to each existing parental superclass according to database structure.

Reactome Pathway (Parental superclass) ID:Description	Number of Overrepresented pathways obtained
R-HSA-1640170:Cell Cycle	43
R-HSA-74160:Gene Expression	38
R-HSA-168256:Immune System	36
R-HSA-1643685:Disease	21
R-HSA-392499:Metabolism of proteins	20
R-HSA-69306:DNA Replication	14
R-HSA-73894:DNA Repair	11
R-HSA-162582:Signal Transduction	8
R-HSA-1430728:Metabolism	5
R-HSA-5357801:Programmed Cell Death	4
R-HSA-1852241:Organelle biogenesis and maintenance	4
R-HSA-2262752:Cellular responses to stress	4
R-HSA-4839726:Chromatin organization	3
R-HSA-109582:Hemostasis	2

A total of 1,232 GO terms from Biological Processes ontology were statistically overrepresented (S7 Table). *Translation* GO term was the most overrepresented based on this list (S3 Fig).

#### Comparison with the literature linked to common inflammatory markers and Th1/Th2 related genes

Regarding the immune system, we compared our results with gene expression studies focused on common inflammatory markers after a single exercise intervention in humans (Table 5) as reviewed by other authors [18]. Different intervention types were considered in this review, but none of them referred to an UMT.

**Table 5:** Differential expressed genes related to common inflammatory markers. List is based on the review presented by other authors [18]. *Gene symbol* and *name* are indicated for each marker in the list. ↓ indicates genes being down-regulated and ↑ genes being up-regulated in *Reg* column. *Prior results* refer to studies where the expression of the specific marker was evaluated in a single exercise-related intervention in humans. Genes are sorted in alphabetical order.

Gene Symbol	Gene name	Reg	Prior results
CCL5 ^(^***^)^	chemokine (C-C motif) ligand 5	↓	[41]
CCR2^(^*^)^, CCR4^(^***^)^	chemokine (C-C motif) receptor 2 and 4	↑ ↓	[17]
CXCL16^(^***^)^	chemokine (C-X-C motif) ligand 16	↑	[42]
GATA3^(^*^)^	GATA binding protein 3	↓	[17]
GPX4^(^***^)^ and GPX7^(^**^)^	glutathione peroxidase 4 and 7	↓ ↓	[43]
HSPA6^(^**^)^	heat shock 70kDa protein 6 (HSP70B')	↑	[44]
IFNGR2^(^***^)^	interferon gamma receptor 2	↑	[45]
IL1RN^(^***^)^ and IL1R1^(^***^)^	interleukin 1 receptor antagonist and type I	↑ ↑	[12]
IL10RB ^(^***^)^ and IL10RB-AS1^(^***^)^	interleukin 10 receptor beta and antisense RNA 1	↑ ↑	[46]^1^
IL1B^(^*^)^	interleukin 1, beta	↑	[12]
IL4R^(^***^)^	interleukin 4 receptor	↑	[17]^2^
IL6R^(^***^)^ and IL6ST^(^***^)^	interleukin 6 receptor and signal transducer	↑ ↓	[47]^3^
MMP9^(^***^)^	matrix metallopeptidase 9	↑	[48]
SOD1^(^**^)^	superoxide dismutase 1, soluble	↓	[49]
TLR2^(^**^)^ and TLR4^(^**^)^	toll-like receptor 2 and 4	↑ ↑	[13]
TGFBR3^(^***^)^^,^ TGFBR3L^(^**^)^ and TGFBRAP1^(^*^)^	transforming growth factor beta receptor III, III-like and associated protein 1	↓ ↑ ↓	[47]
TNF^(^***^)^	tumor necrosis factor	↑	[50]

Results reported on ^1^IL10, ^2^IL4, ^3^IL6.

^(***)^, ^(**)^ and ^(*)^ indicate an adjusted p-value (FDR) < 0.001, < 0.01 and < 0.05 respectively.

We reproduced the same sense of immune imbalance as in [17] where the Th1/Th2 ratio was assessed one week after a marathon race. Although there is a partial overlap in the ranked genes (Table 6) with regard to prior study, we also observed a down-regulation trend in Th1 cytokines and related genes. Of note is the up-regulation of CEBPB which was previously related to Th2 cell response enhancer [51].

**Table 6:** Differential expressed genes related to T-helper 1 and T-helper 2 cells from immune system. Th1 and Th2 cytokines and related genes with differential expression are listed.

Th1/Th2	Regulation	Gene Symbol
Th1 related	Up	TNF^(^***^)^, TLR4^(^**^)^
	Down	STAT1^(^**^)^, STAT4^(^***^)^, TBX21^(^***^)^, CD28^(^***^)^, SOCS1^(^**^)^, SOCS5^(^*^)^
Th2 related	Up	IL1R1^(^***^)^, IL1R2^(^***^)^, STAT6^(^***^)^, CEBPB^(^**^)^, CCR2^(^*^)^, PCGF2^(^*^)^
	Down	NFATC2^(^***^)^, CCL5^(^***^)^, ICOS^(^***^)^, MAF^(^***^)^, CCR4^(^**^)^, GFI1^(^**^)^, GATA3^(^*^)^

^(***)^, ^(**)^ and ^(*)^ indicate an adjusted p-value (FDR) < 0.001, < 0.01 and < 0.05 respectively. Underlined markers are coincident with [17].

#### Identified overrepresented TRs related to hematopoietic cell lineage proliferation, gluconeogenesis and hypoxia situation

A TREA was computed with 4,772 among the 5,084 differential genes which were annotated as TGs to any of the 196 available regulatory elements. Table 7 shows the 27 statistically overrepresented TRs. Only 10 among the 27 ranked TRs had been previously prioritized by the DGEA. From the list, RBL2, RB1 [52] or CTCF [53] are directly involved in chromatin structure modifications. Elements capable of interacting appeared simultaneously. E2F4 binds with high affinity to RBL2 and possibly binds with RB1 which interacts with E2F1 [54]. Eight known transcription factor (TF) families emerged significant (E2F, ETS, FOS, STAT, EGR, GATA, HIF and RUNX). Most of them are related to general processes such as cell cycle, cell proliferation and development. RUNX1, GATA2 and GATA3 act in the development and proliferation of the hematopoietic cell lineage where GATA2 has been considered elsewhere as the master regulator of hematopoietic progenitor cells [55]. TAL1, which collaborates with GATA1, is implicated in several aspects of the final differentiation of red blood cells [56]. HNF4, EGR1, CEBPA and YY1 are TRs described in the gluconeogenesis program in response to a fasting state [57]. HIF1A and EPAS1 are members of the HIF family whose respective signalling pathways were overrepresented. YY1 and EPAS1 are the most selective TFs obtained with, respectively, 90 and 265 out of 23,991 annotated TGs.

**Table 7:** List of the 27 statistically overrepresented transcriptional regulators (TRs) as a response to the intervention. *TR symbol* and *name* are indicated for each TR in the list. *Gene*:*Bg Ratio* indicates the number of target genes regulated by the TR (within the specific list of 4,772 out of 5,084 differential genes which appear in customized TR database obtained from Open Regulatory Annotation database) versus the number of target genes regulated by the TR within the background. Background considers 23,991 genes included in the customized database for TR Enrichment Analysis (TREA). TRs are sorted based on their *adj p-val* (FDR correction) coded as ***<0.001, ** < 0.01 and—in case > 0.05. Last column indicates the *adj p-val* obtained from Differential Gene Expression Analysis (DGEA).

TR Symbol	TR name	Gene:Bg Ratio	adj p-val	adj p-val (DGEA)
E2F4	E2F TF 4, p107/p130-binding	2356:6348	***	-
ETS1	ETS proto-oncogene 1, TF	3453:9824	***	**
RBL2	retinoblastoma-like 2	3642:11908	***	***
SMARCA4	SWI/SNF related, matrix associated, actin dependent regulator of chromatin, subfamily a, member 4	3859:14198	***	-
RB1	retinoblastoma 1	2548:8017	***	-
SPI1	Spi-1 proto-oncogene	1190:2941	***	***
TFAP2C	TF AP-2 gamma (activating enhancer binding protein 2 gamma)	3187:12776	***	-
CEBPA	CCAAT/enhancer binding protein (C/EBP), alpha	2904:11477	***	***
FOS	FBJ murine osteosarcoma viral oncogene homolog	2474:9444	***	***
HNF4A	hepatocyte nuclear factor 4, alpha	2730:10764	***	-
STAT1	signal transducer and activator of transcription 1, 91kDa	2972:12028	***	**
MITF	microphthalmia-associated TF	1763:6480	***	-
CTCF	CCCTC-binding factor (zinc finger protein)	3720:16791	***	-
EGR1	early growth response 1	2780:12041	***	**
VDR	vitamin D (1,25- dihydroxyvitamin D3) receptor	423:1238	***	***
GATA2	GATA binding protein 2	1547:6231	***	-
E2F1	E2F TF 1	1453:5846	***	-
ZNF263	zinc finger protein 263	856:3144	***	**
FOXP1	forkhead box P1	902:3353	***	-
GATA3	GATA binding protein 3	1981:8461	***	**
FOXA1	forkhead box A1	2685:12055	***	-
ESR1	estrogen receptor 1	1656:7051	***	-
TAL1	T-cell acute lymphocytic leukemia 1	864:3489	***	-
HIF1A	hypoxia inducible factor 1, alpha subunit (basic helix-loop-helix TF)	204:692	***	-
RUNX1	runt-related TF 1	656:2692	***	-
EPAS1	endothelial PAS domain protein 1	73:265	**	-
YY1	YY1 TF	30:90	**	-

Abbreviations: TF, transcription factor

### Independent response activity

ICA was computed over a PCA projection at six components determined by the smooth and GCV methods. A matrix with the expression levels of the 5,084 differential genes was used for this purpose. The selection of the number of components was based on the mean error obtained for each number of PCs when applying GCV or smooth method (S4 Fig). First PC is capturing a 52% of data variance and threshold corresponding to 80% of cumulative percentage is achieved by six components (S5 Fig).

ICA decomposed the input expression matrix of 28 array samples × 5,084 differential genes into the mixing matrix A (6 × 5,084) and source matrix S (28 × 6). The mixing matrix contained the weights of 5,804 differential genes for each six independent response blocks to exercise (S6 Fig). A total of 509 main contributors per component were selected corresponding to the highest weight values. S8 Table indicates the number of matches between ICs and respective unique representatives which ranged between 22% (IC6) and 44% (IC3).

#### Dominance of the immune system

First IC was capturing the induced responses both in the innate and in the adaptive immune system according to GEA results conducted over KEGG and Reactome databases (S9 Table and S10 Table respectively). A subset of surface cell markers found in global GEA (CD2, CD3, CD7, CD8, CD14, CD36, CD59, CD116 and CD121) plus new CD28 and CD40LG from *hematopoietic cell lineage* was affected. First line of defense for pathogen recognition arisen with toll-like receptors TLR2, TLR4 and TLR5 in different infectious diseases such as *malaria* (adj pval 0.014), *amoebiasis* (adj pval 0.027) and *legionellosis* (adj pval 0.039) according to GEA over KEGG database. They were also present in Reactome overrepresented pathways *MyD88 deficiency* (adj pval 0.041) and *IRAK4 deficiency* (adj pval 0.048). Ribosome pathway from KEGG was enriched from third IC group of genes, aligned with a considerable number of overrepresented Reactome pathways related to translation. Sixth IC was mainly involved with cell cycle and translation process again according to GEA over Reactome. There were not overrepresented pathways in the rest of ICs.

As a result of TREA, 11 regulator elements were found overrepresented from the group of genes from first IC (S11 Table). Nine of them were already obtained with the global list of differential genes. GATA2 was found in first and third ICs. ETS1 and SMARCA4, also known as BRG1, were found in fourth IC. There were no overrepresented TRs in the rest of ICs.

#### Removal of first line of variance

Previous results provided similar biological insights as the global analysis where all the differential genes were considered. The first line of data variance, accounting for 52% as determined by PC1, featured the immune system response to the intervention. To avoid this, ICA was again computed over the PCA projection at five components after considering the deflationary method over the initial matrix X of 5,084 differential GE levels. Five IC sets were obtained and their 509 main contributors were selected for applying GEA and TREA on each one. The number of matches between them and respective unique elements now ranged between 65% (IC5) and 72% (IC2) (Table 8).

**Table 8:** Main contributors of the independent components (ICs) after applying deflationary method to differential genes expression matrix. Main contributors were selected based on their highest weight values after subtracting one-dimensional data approximation (PC1) to differential genes expression matrix. Those located in the ninth decile were chosen, obtaining a total of 509 genes per IC. Table shows the number of matches between components and the elements that were unique per IC.

Unique genes	#IC	IC1	IC2	IC3	IC4	IC5
342	IC1	509	1	1	91	112
364	IC2		509	142	1	4
358	IC3			509	4	6
358	IC4				509	94
333	IC5					509

#### Terms relative to electron transport chain, a complex signal transduction network and nervous system

**Fig 5:**
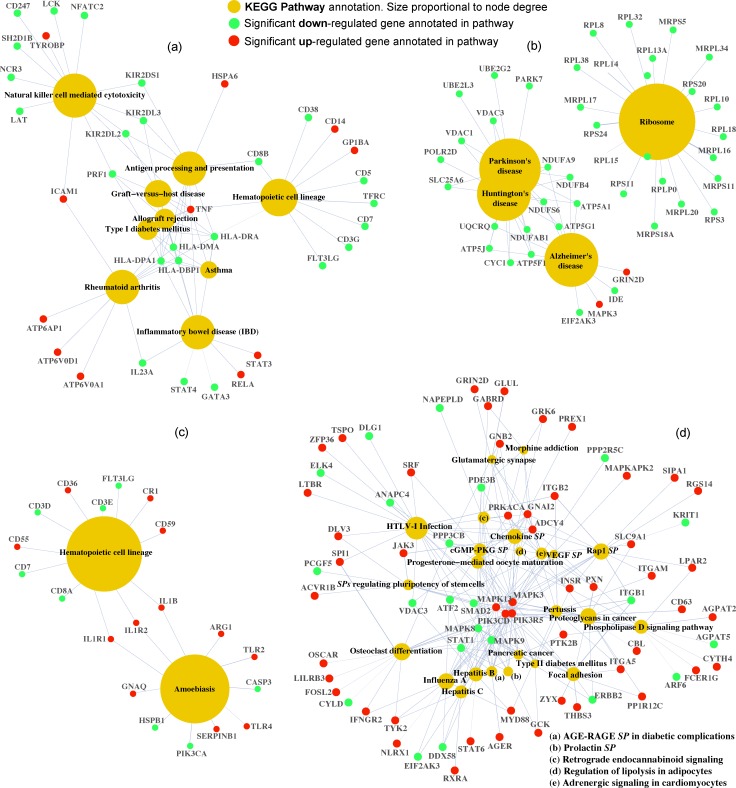
Network of the overrepresented Kyoto Encyclopedia of Genes and Genomes (KEGG) pathways listed in Table 9. Pathways are connected through their differential annotated genes for each Independent Component (IC) after removing first line of variance. (a) IC1 (b) IC2 (c) IC4 and (d) IC3. Pathway’s node size is proportional to the number of annotated genes (node degree). Genes annotated to each pathway are color-coded according to their type of regulation (green codes for down-regulation and red for up-regulation) together with its official gene symbol. SP stands for signalling pathway.

According to GEA results over KEGG (Table 9), first IC elucidated four down-regulated genes responsible for encoding major histocompatibility complex (MHC) class II proteins (HLA-DPA1, HLA-DPB1, HLA-DMA and HLA-DRA) (Fig 5A). These, together with the up-regulated TNF gene, matched in seven out of the nine overrepresented KEGG pathways, related to immune, autoimmune or alloimmune responses. The second IC presented three neurodegenerative diseases: *Parkinson’s*, *Alzheimer’s* and *Huntington’s diseases* (Fig 5B). All of them shared 10 down-regulated genes from the electron transport chain (ETC) in the mitochondrion (Table 10). Genes involved in signal transduction stood out among the 24 KEGG pathways enriched from third IC (Fig 5D). There were two main hubs of signal communication. The up-regulated MAPK3, MAPK13, PIK3R5, PIK3CD genes and the down-regulated MAPK8 and MAPK9 genes characterized one hub. Among them, MAPK3, PIK3R5 and PIK3CD were common for 20 out of the 24 pathways. The other hub was featured by the up-regulated PRKACA and ADCY4, related to cAMP second messengers, and GNAI2 gene. Two pathways associated with the nervous system, *retrograde endocannabinoid signalling* and *morphine addiction*, showed up-regulation in most of their annotated differential genes with a 70% and a 85.7% respectively. GABRD gene which encodes for a neurotransmitter GABA receptor was one of these up-regulated genes. OSCAR and AGER genes, both up-regulated, were respectively specific elements for *osteoclast differentiation* and *AGE-RAGE signalling pathway in diabetic complications* pathways. Five infectious diseases were overrepresented, four of them being of viral origin: *HTLV-I infection*, *hepatitis C*, *hepatitis B* and *influenza A*. The up-regulated SERPINB1 gene was annotated to, inter alia, the overrepresented *amoebiasis* KEGG pathway from fourth IC (Fig 5C). The role of this gene has been previously related to the mitigation of inflammation in pulmonary influenza infections [58]. Genes encoding ribosomal proteins, including mitochondrial ribosomal proteins, characterized the fifth IC.

**Table 9:** List of the statistically overrepresented Kyoto Encyclopedia of Genes and Genomes (KEGG) pathways obtained for each independent component (IC) after removing first line of variance. *KEGG pathway ID* and *description* is enclosed in the table. Pathway’s *main category* and *subcategory* are shown. *Gene*:*Bg Ratio* indicates the number of genes annotated to a pathway within the specific list of differential genes among the 509 major contributors that are included in the database (i.e. 201 for IC1) versus the number of genes annotated to a pathway within the background. Background considers all differential genes included in KEGG database which corresponds to 1905 elements among 5084 differential genes. Pathways are sorted based on their *adj p-val* (FDR correction) coded as *** < 0.001, ** < 0.01 and * < 0.05. *Up-reg* indicates the percentage of differential genes annotated to the specific pathway being up-regulated.

#IC	KEGG Pathway ID:Description	Main Category—Subcategory	Gene: Bg Ratio	adj p-val	Up-reg [%]
IC1	hsa05332:Graft-versus-host disease	HD—Immune diseases	8/201:17	*	12.5
	hsa05323:Rheumatoid arthritis	HD—Immune diseases	10/201:26	*	50
	hsa05321:Inflammatory bowel disease (IBD)	HD—Immune diseases	10/201:29	*	30
	hsa04640:Hematopoietic cell lineage	OS—Immune system	11/201:36	*	27.3
	hsa05330:Allograft rejection	HD—Immune diseases	6/201:13	*	16.7
	hsa04612:Antigen processing and presentation	OS—Immune system	10/201:33	*	20
	hsa04940:Type I diabetes mellitus	HD—Endocrine and metabolic diseases	6/201:14	*	16.7
	hsa05310:Asthma	HD—Immune diseases	5/201:10	*	20
	hsa04650:Natural killer cell mediated cytotoxicity	OS—Immune system	13/201:52	*	23.1
IC2	hsa05012:Parkinson's disease	HD—Neurodegenerative diseases	16/188:46	***	0
	hsa03010:Ribosome	GIP—Translation	20/188:75	**	0
	hsa05010:Alzheimer's disease	HD—Neurodegenerative diseases	14/188:57	*	14.3
	hsa05016:Huntington's disease	HD—Neurodegenerative diseases	14/188:57	*	0
IC3	hsa04723:Retrograde endocannabinoid signaling	OS—Nervous system	10/204:25	*	70
	hsa04914:Progesterone-mediated oocyte maturation	OS—Endocrine system	11/204:30	*	63.6
	hsa05133:Pertussis	HD—Infectious diseases: Bacterial	10/204:28	*	70
	hsa04923:Regulation of lipolysis in adipocytes	OS—Endocrine system	7/204:16	*	85.7
	hsa04380:Osteoclast differentiation	OS—Development	15/204:59	*	66.7
	hsa04015:Rap1 signaling pathway	EIP—Signal transduction	14/204:55	*	85.7
	hsa05166:HTLV-I infection	HD—Infectious diseases: Viral	20/204:93	*	60
	hsa05160:Hepatitis C	HD—Infectious diseases: Viral	11/204:38	*	54.5
	hsa04072:Phospholipase D signaling pathway	EIP—Signal transduction	12/204:44	*	83.3
	hsa05032:Morphine addiction	HD—Substance dependence	7/204:18	*	85.7
	hsa04724:Glutamatergic synapse	OS—Nervous system	8/204:24	*	87.5
	hsa04917:Prolactin signaling pathway	OS—Endocrine system	8/204:24	*	62.5
	hsa04022:cGMP-PKG signaling pathway	EIP—Signal transduction	11/204:41	*	63.6
	hsa04370:VEGF signaling pathway	EIP—Signal transduction	7/204:20	*	85.7
	hsa04930:Type II diabetes mellitus	HD—Endocrine and metabolic diseases	7/204:20	*	71.4
	hsa05205:Proteoglycans in cancer	HD—Cancers: Overview	14/204:61	*	78.6
	hsa04510:Focal adhesion	CP—Cellular community	12/204:49	*	66.7
	hsa05212:Pancreatic cancer	HD—Cancers: Specific Types	8/204:26	*	37.5
	hsa04062:Chemokine signaling pathway	OS—Immune system	13/204:56	*	92.3
	hsa04550:Signaling pathways regulating pluripotency of stem cells	CP—Cellular community	9/204:32	*	77.8
	hsa04933:AGE-RAGE signaling pathway in diabetic complications	HD—Endocrine and metabolic diseases	9/204:32	*	55.6
	hsa05161:Hepatitis B	HD—Infectious diseases: Viral	12/204:50	*	50
	hsa04261:Adrenergic signaling in cardiomyocytes	OS—Circulatory system	10/204:38	*	80
	hsa05164:Influenza A	HD—Infectious diseases: Viral	14/204:63	*	57.1
IC4	hsa05146:Amoebiasis	HD—Infectious diseases: Parasitic	11/204:27	**	72.7
	hsa04640:Hematopoietic cell lineage	OS—Immune system	12/204:36	*	58.3
IC5	hsa03010:Ribosome	GIP—Translation	18/176:75	*	0

Abbreviations: GIP, Genetic Information Processing; OS, Organismal System; HD, Human Diseases; CP, Cellular Processes; EIP, Environmental Information Processing.

**Table 10:** Down-regulated genes from the electron transport chain as a response to the intervention.

Gene Symbol	Gene name	ETC Complex
NDUFA9^(^***^)^	NADH dehydrogenase (ubiquinone) 1 alpha subcomplex, 9, 39kDa	I
NDUFAB1^(^***^)^	NADH dehydrogenase (ubiquinone) 1, alpha/beta subcomplex, 1, 8kDa	I
NDUFS6^(^**^)^	NADH dehydrogenase (ubiquinone) Fe-S protein 6, 13kDa (NADH-coenzyme Q reductase)	I
NDUFB4^(^*^)^	NADH dehydrogenase (ubiquinone) 1 beta subcomplex, 4, 15kDa	I
CYC1^(^**^)^	cytochrome c-1	III
UQCRQ^(^**^)^	ubiquinol-cytochrome c reductase, complex III subunit VII, 9.5kDa	III
ATP5A1^(^***^)^	ATP synthase, H+ transporting, mitochondrial F1 complex, alpha subunit 1, cardiac muscle	V
ATP5G1^(^***^)^	ATP synthase, H+ transporting, mitochondrial Fo complex, subunit C1 (subunit 9)	V
ATP5J ^(^**^)^	ATP synthase, H+ transporting, mitochondrial Fo complex, subunit F6	V
ATP5F1^(^**^)^	ATP synthase, H+ transporting, mitochondrial Fo complex, subunit B1	V

^(***)^, ^(**)^ and ^(*)^ indicate an adjusted p-value (FDR) < 0.001, < 0.01 and < 0.05 respectively. ETC, electron transport chain.

A summary of the GEA results over Reactome is included in S12 Table. Overrepresented pathways were only found for second and fifth ICs. In line with KEGG results, ETC was also shown in 2 among the 31 enriched pathways in the second IC: *the citric acid (TCA) cycle and respiratory electron transport* and the *respiratory electron transport*, *ATP synthesis by chemiosmotic coupling*, *and heat production by uncoupling proteins* pathways.

#### Overrepresented TRs among ICs are aligned with pathway enrichment results

A list of 19 TRs was found overrepresented in IC2, IC3 and IC4 as a result of TREA (Table 11). Most of them, 17 out of the 19, were already prioritized when considering a global response (Table 7). In this case, ETS1 and E2F4 transcription factors were enriched in the second IC. E2F4 belong to E2F family which is known by its dual role in cell proliferation and its contribution to cell death in response to cell stress [59]. The third IC showed five enriched TRs (EGR1, VDR, ZNF263, TFAP2C and CTCF) where, as best we know, the last three have an unspecific TR role. EGR1 was connected to MAPK and vascular endothelial growth factor (VEGF) signalling, both highlighted GEA results for IC3 (Table 9), when studying the relationship between insulin sensitivity and exercise-induced gene expression [60]. The VDR gene is known to be connected to bone homeostasis [61] which is compatible with the presence of osteoclast differentiation pathway (Table 7 –IC3). From fourth IC, TP53 and SOX2 genes were the new hits found.

**Table 11:** List of the statistically overrepresented transcriptional regulators (TRs) obtained per independent component (IC) after removing first line of variance. Three ICs (IC2, IC3 and IC4) among the computed five components show enriched TRs. *TR symbol* and *name* are indicated for each TR in the list. *Gene*:*Bg Ratio* indicates the number of target genes (TGs) regulated by the specific TR among the 509 major contributor genes versus the number of TGs regulated by the TR within the background. Only those major contributors that appear in the customized TR database obtained from Open Regulatory Annotation (ORA) database per IC are considered (i.e. for IC2, 489 out of the 509 contributors). Background considers 4,772 genes included in the customized ORA database for TR Enrichment Analysis (TREA) among 5,084 differential genes. TRs are sorted based on their *adj p-val* (FDR correction) coded as *** < 0.001, ** < 0.01, * < 0.05 and—in case > 0.05. Last column indicates the *adj p-val* obtained from Differential Gene Expression Analysis (DGEA).

#IC	TR symbol	TR name	Gene:Bg Ratio	adj p-val	adj p-val (DGEA)
IC2	ETS1	ETS proto-oncogene 1, TF	391/489:3453	**	***
	E2F4	E2F TF 4, p107/p130-binding	275/489:2356	**	-
IC3	EGR1	Early growth response 1	350/493:2780	***	***
	VDR	Vitamin D (1,25- dihydroxyvitamin D3) receptor	81/493:423	***	**
	ZNF263	Zinc finger protein 263	137/493:856	***	*
	TFAP2C	TF AP-2 gamma (activating enhancer binding protein 2 gamma)	366/493:3187	***	-
	CTCF	CCCTC-binding factor (zinc finger protein)	409/493:3720	**	-
IC4	GATA2	GATA binding protein 2	211/470:1547	***	-
	FOXA1	Forkhead box A1	319/470:2685	***	-
	GATA3	GATA binding protein 3	246/470:1981	***	*
	SMARCA4	SWI/SNF related, matrix associated, actin dependent regulator of chromatin, subfamily a, member 4	416/470:3859	***	-
	SPI1	Spi-1 proto-oncogene	160/470:1190	***	***
	TAL1	T-cell acute lymphocytic leukemia 1	121/470:864	***	-
	TP53	Tumor protein p53	80/470:548	***	-
	CEBPA	CCAAT/enhancer binding protein (C/EBP), alpha	321/470:2904	**	**
	STAT1	Signal transducer and activator of transcription 1, 91kDa	326/470:2972	**	**
	CTCF	CCCTC-binding factor (zinc finger protein)	392/470:3720	**	-
	ESR1	Estrogen receptor 1	193/470:1656	**	-
	SOX2	SRY-box 2	97/470:746	**	-

Abbreviations: TF, transcription factor

## Discussion and conclusions

Previous studies have accumulated evidence about the health risk reduction as a result of moderate physical activity [1]. Nevertheless, an U-curve pattern has been previously described when considering the effect of high intensity and prolonged exercise over cardiovascular [3] or URTI [16] risks. In this sense, an UMT is of interest due to its extreme conditions [5] and its consequences on the whole body homeostasis. To our knowledge, the present study is the first genome-wide investigation aiming an expression profiling in response to a UMT race.

Our results show that gene expression is heavily impacted by the intervention based on the 5,084 protein-coding genes, among 23,557 initially tested, with significant differential expression. The global gene enrichment analysis reveals extensive alterations in human biology mainly concentrated around the immune system, infectious diseases and genetic information processing.

A 36% of the enriched infectious diseases terms (Table 3) are caused by parasitic (*Toxoplasmosis*) and viral pathogens (*Epstein-Barr virus infection*, *Herpes simplex infection* and *Influenza A*) associated with URTI [62], An additional 27% implicates pathogens responsible of other respiratory infections such *Legionellosis*, *Measles* and *Tuberculosis*, the latter primarily attacking the lungs, while the rest were unrelated respiratory infections. These results do not necessarily imply that subjects presented a particular infection, but its genetic mechanisms triggered by the strenuous exercise.

We interpret protein synthesis as repressed based on the systematic down-regulation of the genes annotated to the related intracellular processes. This response is compatible with two opposite situations: the negative energy balance due to the high-demanding exercise [63] and, as defined by other authors, the maintenance of protein levels is a bioenergetically expensive process [64]. In a similar experiment using muscle biopsy samples, authors found an activation of muscle protein degradation in addition to muscle protein repression [65]. The autophagy-lysosomal and the ubiquitin proteasome pathways (UPP) mainly control protein degradation in skeletal muscle [66]. We report an overrepresentation of the *Lysosome* pathway with a general up-regulation of up to 65% of its annotated differential genes. Controversially, overrepresented pathways related to UPP clearly emerged down-regulated, *Ubiquitin mediated proteolysis* and *Proteasome*, with the 78% and 91% of their annotated differential genes respectively.

HIF-1 signalling pathway enrichment is aligned with the TREA results where EPAS1 (aka HIF-2α) and HIF-1A were found. We identified the up-regulation of genes related to the increase oxygen delivery (TIMP-1, HMOX-1), oxygen consumption reduction (HK, ALDOA and PFK2) and associated TR (HIF-1β aka ARNT). In human skeletal muscle studies, HIF-1 has been held to be responsible for, among other functions, a reduction in mitochondrial activity [20] and VEGF regulation [67]. Its activation has been previously reported after a single exercise [68]. On the other hand, the EPAS1 gene is a TF that plays a key role in the HIF pathway by activating genes in response to hypoxia [69], specifically those involved in erythropoiesis and angiogenesis [70]. While several studies have evaluated the influence of EPAS1 genetic variants in individual aerobic capacity [70] and athletic performance [71]; to our knowledge no specific studies have explored the EPAS1 response to exercise from a transcriptomics approach. Additionally, there is prior evidence of the collaboration of ETS1, another overrepresented TF, with HIF-1 in regulating hypoxia-inducible genes in pathological situations [72].

ICA identified further biological pathways including key alteration in mitochondrial activity and endocannabinoid signalling. Several genes from the ETC were systematically down-regulated as a obtained from the GEA applied to the ICs (Table 10). Most of them (NDUFA9, NDUFAB1, CYC1, UQCRQ and ATP5A1) are reported as a direct effect of ETS1 in cancer cells in its role of mitochondrial stress and dysfunction regulation [73]. TP53 gene was one of the additional transcriptional regulators retrieved after applying ICA. TP53 stands as an stress sensor of the cell such as oxidative stress, hypoxia and nutrient depravation [74], signals compatible with the experiment. Additionally, the TP53 gene has been related to the regulation of mitochondrial respiration [75] and possible exercise-induced mitochondrial biogenesis [2,76] through interactions with TFAM in the mitochondria. However, we have not observed any differential expression in TFAM. With regard to the endocannabinoid-signalling pathway, recent studies in mice describe the so called “runner’s high” dependence on the endocannabinoid system in response to wheel running [77] and how this exercise-induced effect is intensity-modulated in humans [78,79].

The current study has, however, certain limitations. First, the small sample size could limit the results validation in newer cohorts. However, the findings are consistent with existing literature in exercise-related studies. Secondly, the physical effort of each runner may be heterogeneous for the same completed distance. Nevertheless, this feature may be difficult to include in the linear regression model beyond the runner’s subjective perception.

In conclusion, the present study points to almost one fourth of all protein-coding genes affected by running an UMT, with a substantial number of human biology pathways overrepresented. In agreement with prior exercise-related studies, the global physiological approach is predominantly associated with immune system, infectious diseases and genetic information processing. The independent activity approach revealed additional pathways beyond the abovementioned which will require tailored investigations in larger sample sizes. Biological pathways and transcriptional regulators overrepresentation analysis offered a complementary interpretation of the results.

## Supporting information

S1 Fig Quality control–PCA over the expression values of the pre-processed microarray data from the initial 29 samples. (PDF)

S2 Fig Overrepresented hematopoietic cell lineage extracted from KEGG pathway database with differential genes highlighted. (PDF)

S3 Fig Summary of the 1232 statistically overrepresented GO terms from Biological Processes ontology. (PDF)

S4 Fig Estimation of the optimal number of components in PCA with GCV and smooth methods. (PDF)

S5 Fig Cumulative percentage of variance in PCA computed over the expression matrix of 5,084 differential genes. (PDF)

S6 Fig Histogram of each k^th^ row of the mixing matrix A representing the weights of the 5,084 differential genes. (PDF)

S1 Table Software and package versions used in the study. (PDF)

S2 Table List of 5,499 differential expressed transcript clusters with single gene annotation. (CSV)

S3 Table List of 475 differential expressed transcript clusters with multiple gene annotations. (CSV)

S4 Table List of 35 differential expressed transcript clusters related to gender contribution. (CSV)

S5 Table Detailed list of the 42 overrepresented pathways from KEGG as a result of GEA. (CSV)

S6 Table Detailed list of the 193 overrepresented pathways from Reactome as a result of GEA. (CSV)

S7 Table Detailed list of the 1233 overrepresented GO terms from GO–Biological Processes ontology as a result of GEA. (CSV)

S8 Table Number of main contributors to each IC based on their highest weight values. (PDF)

S9 Table List of the statistically overrepresented KEGG pathways obtained per IC after ICA. (PDF)

S10 Table List of the statistically overrepresented Reactome pathways obtained per IC after ICA. (PDF)

S11 Table List of the statistically overrepresented transcriptional regulators (TRs) obtained per IC after ICA. (PDF)

S12 Table List of the statistically overrepresented Reactome pathways obtained for IC2 and IC5 after removing first line of variance. (PDF)
