# Supplementary material for: Affected pathways and transcriptional regulators in gene expression response to an ultra-marathon trail: Global and independent activity approaches
Source: PLoS One. 2017 Oct 13;12(10):e0180322. doi: 10.1371/journal.pone.0180322 (PMC5640184; doi:10.1371/journal.pone.0180322)
Supplement: S1 Fig — (PDF) [file pone.0180322.s001.pdf]

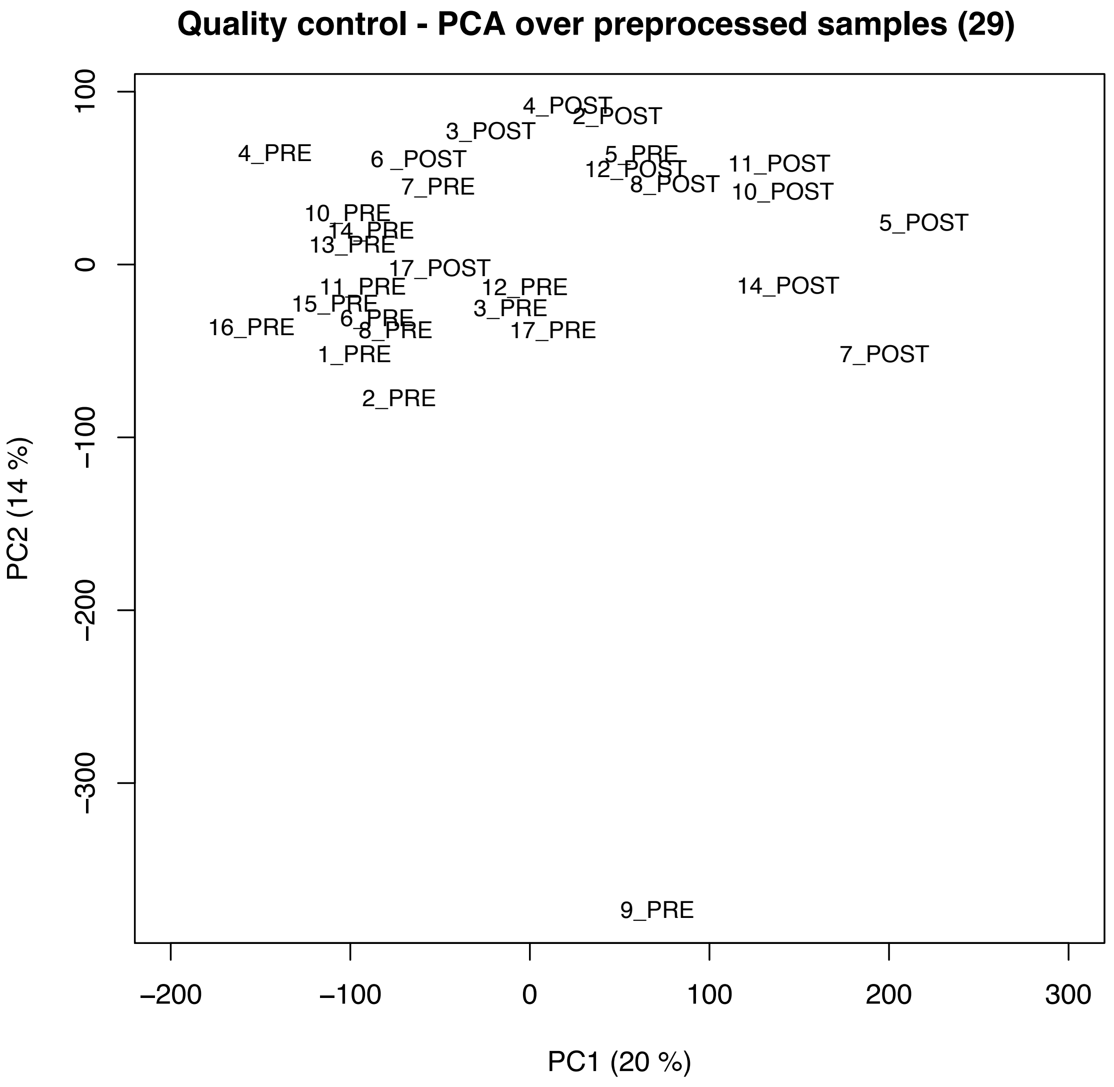

S1 Fig. Quality control – PCA over the expression values of the pre-processed microarray data from the initial 29 samples. PCA over 53,617 TCs expression values included in HuGene20st microarray. First (abscissa) and second principal components (ordinate) are shown. They are capturing 20% (PC1) and 14% (PC2) of the total data variance. Sample identified as 9_PRE clearly shows a distinct pattern compared to the rest of arrays so it was excluded for downstream analysis.
