# Supplementary material for: Affected pathways and transcriptional regulators in gene expression response to an ultra-marathon trail: Global and independent activity approaches
Source: PLoS One. 2017 Oct 13;12(10):e0180322. doi: 10.1371/journal.pone.0180322 (PMC5640184; doi:10.1371/journal.pone.0180322)
Supplement: S2 Fig — (PDF) [file pone.0180322.s002.pdf]

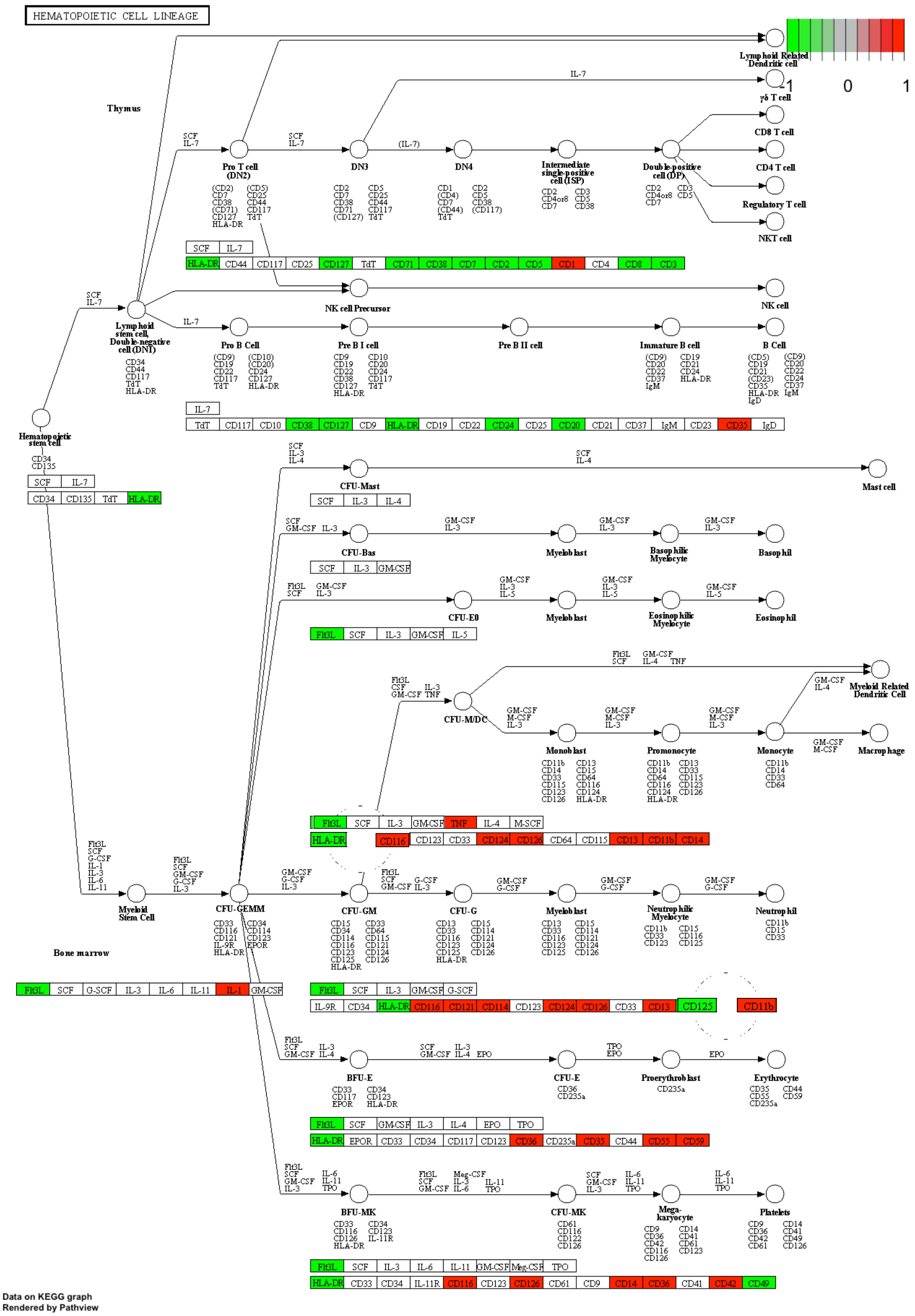

S2 Fig. Overrepresented hematopoietic cell lineage extracted from KEGG Pathway database with differential genes highlighted. This KEGG pathway (ID hsa04640) is ranked according to results from GEA. Differential genes are coloured in red or green if they are up- or down-regulated respectively. Reprinted from [1,2,3] under a CC BY license, with permission from *Kanehisa Laboratories*, original copyright 2016.

[1] Kanehisa M, Goto S. KEGG: kyoto encyclopedia of genes and genomes. Nucleic Acids Res 2000;28:27–30.

[2] Kanehisa M., Tanabe, M., Sato, Y., and Morishima, K. KEGG: new perspectives on genomes, pathways, diseases and drugs. Nucleic Acids Res 2017; 45, D353-D361.

[3] Kanehisa, M., Sato, Y., Kawashima, M., Furumichi, M., and Tanabe, M. KEGG as a reference resource for gene and protein annotation. Nucleic Acids Res 2016; 44, D457-D462.
