# Supplementary material for: Affected pathways and transcriptional regulators in gene expression response to an ultra-marathon trail: Global and independent activity approaches
Source: PLoS One. 2017 Oct 13;12(10):e0180322. doi: 10.1371/journal.pone.0180322 (PMC5640184; doi:10.1371/journal.pone.0180322)
Supplement: S3 Fig — (PDF) [file pone.0180322.s003.pdf]

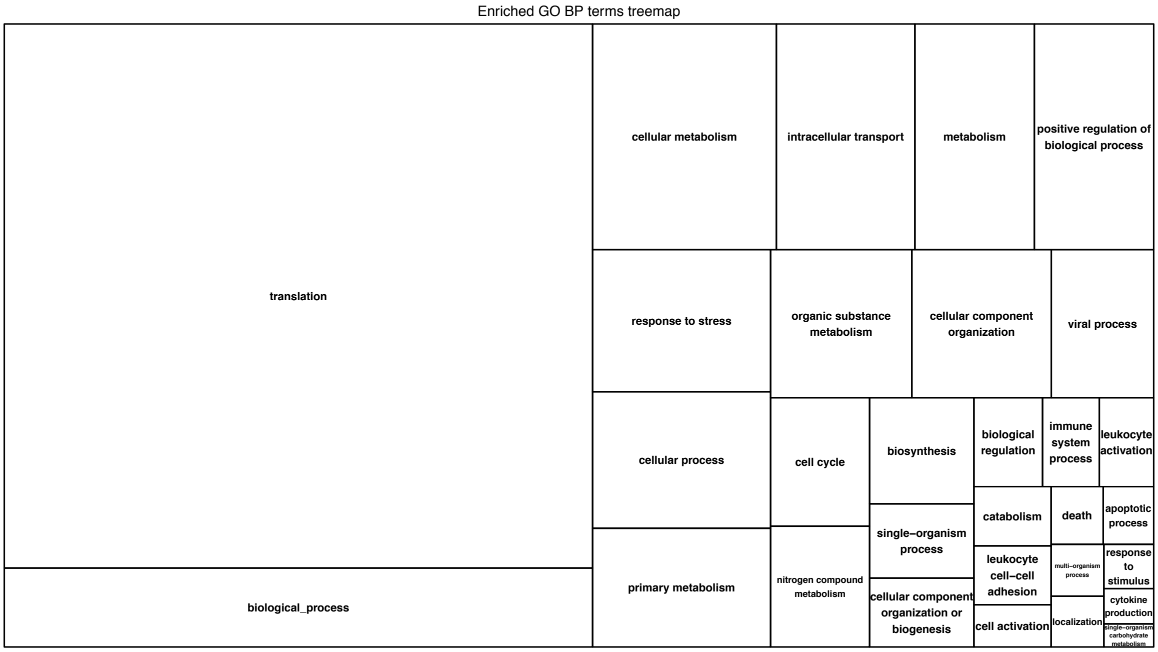

S3 Fig. Summary of the 1232 statistically overrepresented GO terms from Biological Processes ontology. Obtained terms are grouped and annotated to their representative based on a semantic similarity measure through REVIGO web server [1] using default parameters. Area of each representative rectangle is inversely proportional to the p-value obtained in the hypergeometric test. The treemap was generated with *treemap* package [2].

[1] F. Supek, M. Bošnjak, N. Škunca, and T. Šmuc, "REVIGO summarizes and visualizes long lists of gene ontology terms.," *PLoS One*, vol. 6, no. 7, p. e21800, Jan. 2011.

[2] M. Tennekes, "treemap: Treemap Visualization. R package version 2.4-1." 2016.
