# Supplementary material for: Affected pathways and transcriptional regulators in gene expression response to an ultra-marathon trail: Global and independent activity approaches
Source: PLoS One. 2017 Oct 13;12(10):e0180322. doi: 10.1371/journal.pone.0180322 (PMC5640184; doi:10.1371/journal.pone.0180322)
Supplement: S4 Fig — (PDF) [file pone.0180322.s004.pdf]

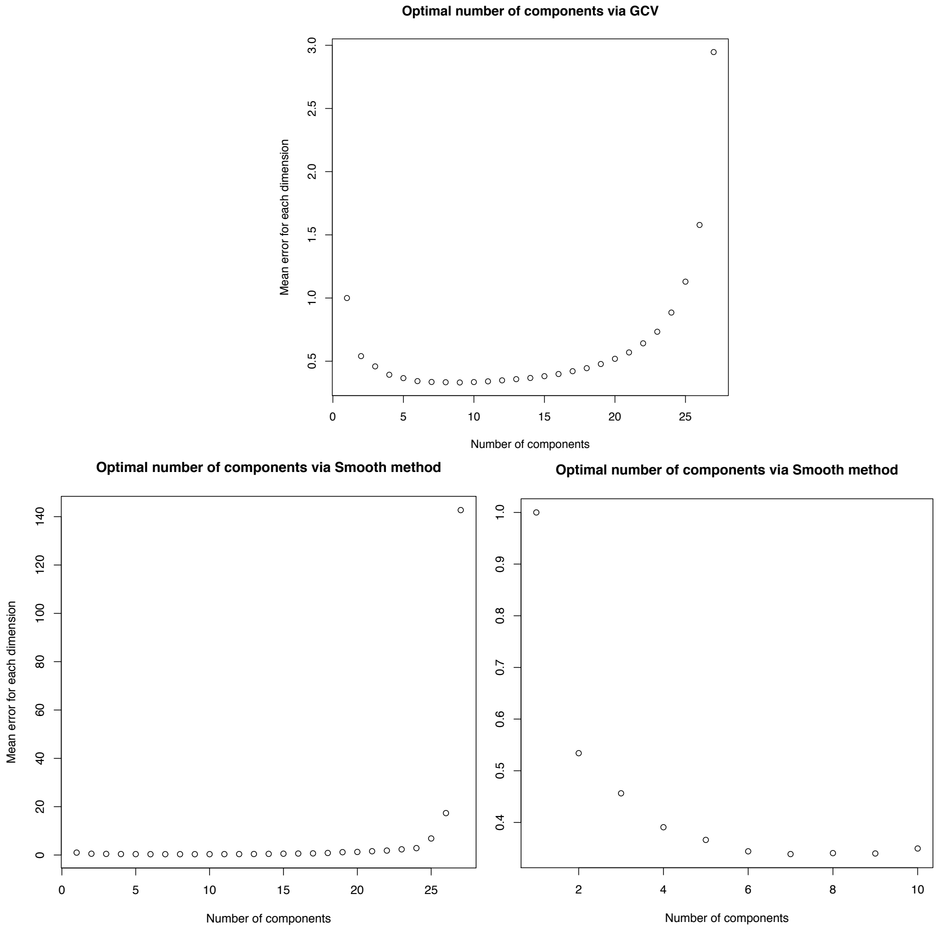

S4 Fig. Estimation of the optimal number of components in PCA with GCV and smooth methods. The expression matrix of 28 samples x 5,084 differential genes is considered for this purpose. Mean error for each dimension tested is shown for all components with GCV (top) and smooth methods (bottom). Results from smooth methods are zoom in on first ten components (bottom right).
