# Supplementary material for: Affected pathways and transcriptional regulators in gene expression response to an ultra-marathon trail: Global and independent activity approaches
Source: PLoS One. 2017 Oct 13;12(10):e0180322. doi: 10.1371/journal.pone.0180322 (PMC5640184; doi:10.1371/journal.pone.0180322)
Supplement: S5 Fig — (PDF) [file pone.0180322.s005.pdf]

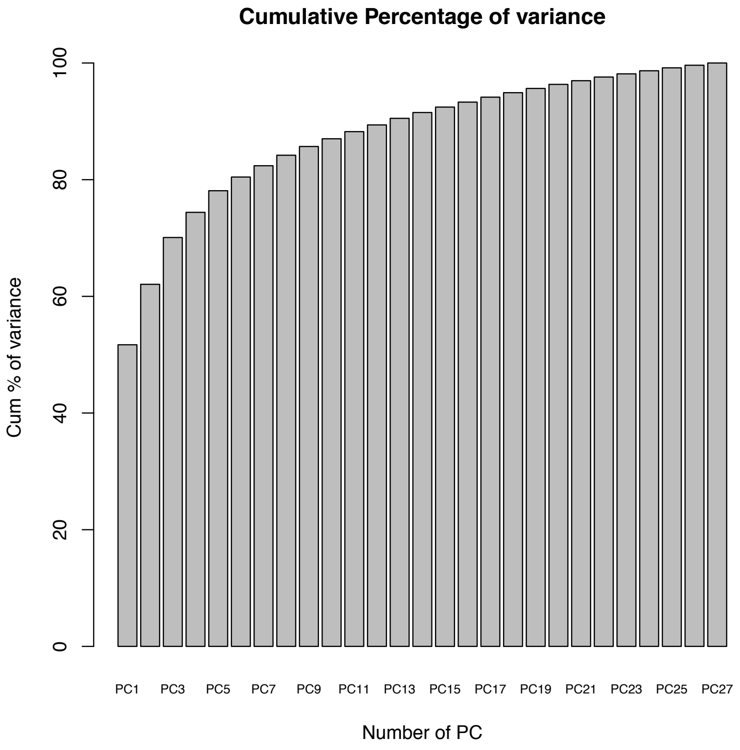

S5 Fig. Cumulative percentage of variance in PCA computed over the expression matrix of 5,084 differential genes.
