# Supplementary material for: Affected pathways and transcriptional regulators in gene expression response to an ultra-marathon trail: Global and independent activity approaches
Source: PLoS One. 2017 Oct 13;12(10):e0180322. doi: 10.1371/journal.pone.0180322 (PMC5640184; doi:10.1371/journal.pone.0180322)
Supplement: S1 Table — (PDF) [file pone.0180322.s007.pdf]

**S1 Table. Software and package versions used in the study**.

| **Software/Package** | **Version** |
|---|---|
| R software | v3.2.0 |
| BioConductor | v3.1 |
| *clusterProfiler* | v2.2.3 |
| *FactoMineR* | v1.31.4 |
| *fastICA* | v1.2-0 |
| *genefilter* | v1.50.0 |
| *gplots* | v2.17.0 |
| *hugene20sttranscriptcluster.db* | v8.3.1 |
| *limma* | v3.24.13 |
| *oligo* | v1.32.0 |
