# Supplementary material for: Affected pathways and transcriptional regulators in gene expression response to an ultra-marathon trail: Global and independent activity approaches
Source: PLoS One. 2017 Oct 13;12(10):e0180322. doi: 10.1371/journal.pone.0180322 (PMC5640184; doi:10.1371/journal.pone.0180322)
Supplement: S8 Table — (PDF) [file pone.0180322.s014.pdf]

S8 Table. Number of main contributors to each IC based on their highest weight values. Those located in the ninth decile were chosen, obtaining a total of 509 genes per IC. Table shows the number of matches between components and the elements that were unique per IC.

Unique genes	#IC	IC1	IC2	IC3	IC4	IC5	IC6
190	**IC1**	509	124	179	75	68	99
225	**IC2**		509	84	108	102	94
206	**IC3**			509	50	101	83
187	**IC4**				509	136	204
163	**IC5**					509	209
111	**IC6**						509
