# Supplementary material for: Affected pathways and transcriptional regulators in gene expression response to an ultra-marathon trail: Global and independent activity approaches
Source: PLoS One. 2017 Oct 13;12(10):e0180322. doi: 10.1371/journal.pone.0180322 (PMC5640184; doi:10.1371/journal.pone.0180322)
Supplement: S9 Table — (PDF) [file pone.0180322.s015.pdf]

S9 Table. List of the statistically overrepresented KEGG pathways obtained per IC after ICA. Only two ICs, IC1 and IC3, among the computed six components shown enriched pathways. ID and description pathway is enclosed in the table. *Gene Ratio* indicates the number of genes annotated to a pathway within the specific list of differential genes among the 509 major contributors that are included in the database (203 for IC1 and 220 for IC3). *Bg Ratio* refers to the number of genes annotated to a pathway within the background (all differential genes included in the database which is a total of 1905 elements among 5084). Pathways are sorted based on the adj p-value obtained (FDR).

#IC	KEGG Pathway ID:Description	GeneRatio	BgRatio	adj p-val
IC1	hsa04640: Hematopoietic cell lineage	17/203	36/1905	<0.001
	hsa04650: Natural killer cell mediated cytotoxicity	16/203	52/1905	0.004
	hsa04660: T cell receptor signaling pathway	16/203	52/1905	0.004
	hsa05332: Graft-versus-host disease	8/203	17/1905	0.008
	hsa04514: Cell adhesion molecules (CAMs)	13/203	41/1905	0.008
	hsa05144: Malaria	8/203	19/1905	0.014
	hsa04060: Cytokine-cytokine receptor interaction	13/203	45/1905	0.016
	hsa05320: Autoimmune thyroid disease	6/203	13/1905	0.027
	hsa05330: Allograft rejection	6/203	13/1905	0.027
	hsa05146: Amoebiasis	9/203	27/1905	0.027
	hsa04610: Complement and coagulation cascades	5/203	10/1905	0.039
	hsa05134: Legionellosis	9/203	29/1905	0.039
	hsa05150: Staphylococcus aureus infection	7/203	19/1905	0.039
IC3	hsa03010: Ribosome	36/220	75/1905	<0.001
	hsa04640: Hematopoietic cell lineage	13/220	36/1905	0.010
	hsa04064: NF-kappa B signaling pathway	13/220	39/1905	0.017
