# Supplementary material for: Affected pathways and transcriptional regulators in gene expression response to an ultra-marathon trail: Global and independent activity approaches
Source: PLoS One. 2017 Oct 13;12(10):e0180322. doi: 10.1371/journal.pone.0180322 (PMC5640184; doi:10.1371/journal.pone.0180322)
Supplement: S10 Table — (PDF) [file pone.0180322.s016.pdf]

S10 Table. List of the statistically overrepresented Reactome pathways obtained per IC after ICA. Only IC1, IC3 and IC6 among the computed six components shown enriched pathways. ID and description pathway is enclosed in the table. *Gene Ratio* indicates the number of genes annotated to a pathway within the specific list of differential genes among the 509 major contributors that are included in the database (185 for IC1, 229 for IC3 and 171 for IC6). *Bg Ratio* refers to the number of genes annotated to a pathway within the background (all differential genes included in the database which is a total of 1895 elements among 5084). Pathways are sorted based on the adj p-value obtained (FDR).

#IC	ID	Description	GeneRatio	BgRatio	p.adj
IC1	198933	Immunoregulatory interactions between a Lymphoid and a non-Lymphoid cell	17/185	34/1895	<0.001
	168256	Immune System	62/185	361/1895	<0.001
	1280218	Adaptive Immune System	34/185	165/1895	0.001
	373076	Class A/1 (Rhodopsin-like receptors)	16/185	52/1895	0.002
	500792	GPCR ligand binding	17/185	59/1895	0.002
	202433	Generation of second messenger molecules	8/185	17/1895	0.006
	388396	GPCR downstream signaling	20/185	95/1895	0.032
	202403	TCR signaling	10/185	32/1895	0.032
	168249	Innate Immune System	35/185	211/1895	0.032
	2022870	Chondroitin sulfate biosynthesis	5/185	9/1895	0.034
	5602498	MyD88 deficiency (TLR2/4)	4/185	6/1895	0.041
	5603041	IRAK4 deficiency (TLR2/4)	4/185	6/1895	0.041
	202430	Translocation of ZAP-70 to Immunological synapse	5/185	10/1895	0.048
IC3	156842	Eukaryotic Translation Elongation	33/229	54/1895	<0.001
	192823	Viral mRNA Translation	32/229	51/1895	<0.001
	72764	Eukaryotic Translation Termination	32/229	51/1895	<0.001
	156902	Peptide chain elongation	32/229	53/1895	<0.001
	975956	Nonsense Mediated Decay (NMD) independent of the Exon Junction Complex (EJC)	32/229	53/1895	<0.001
	72689	Formation of a pool of free 40S subunits	34/229	61/1895	<0.001
	927802	Nonsense-Mediated Decay (NMD)	33/229	58/1895	<0.001
	975957	Nonsense Mediated Decay (NMD) enhanced by the Exon Junction Complex (EJC)	33/229	58/1895	<0.001
	156827	L13a-mediated translational silencing of Ceruloplasmin expression	35/229	66/1895	<0.001
	157279	3' -UTR-mediated translational regulation	35/229	66/1895	<0.001
	72706	GTP hydrolysis and joining of the 60S ribosomal subunit	35/229	67/1895	<0.001
	72613	Eukaryotic Translation Initiation	35/229	69/1895	<0.001
	72737	Cap-dependent Translation Initiation	35/229	69/1895	<0.001
	168255	Influenza Life Cycle	33/229	65/1895	<0.001
	168254	Influenza Infection	33/229	66/1895	<0.001
	168273	Influenza Viral RNA Transcription and Replication	32/229	63/1895	<0.001
	1799339	SRP-dependent cotranslational protein targeting to membrane	32/229	66/1895	<0.001
	72766	Translation	36/229	87/1895	<0.001
	72649	Translation initiation complex formation	19/229	38/1895	<0.001
	72662	Activation of the mRNA upon binding of the cap-binding complex and eIFs, and subsequent binding to 43S	19/229	38/1895	<0.001
	72702	Ribosomal scanning and start codon recognition	19/229	38/1895	<0.001
	72695	Formation of the ternary complex, and subsequently, the 43S complex	18/229	35/1895	<0.001
	5663205	Infectious disease	43/229	159/1895	<0.001
	1643685	Disease	55/229	244/1895	<0.001
	1489509	DAG and IP3 signaling	5/229	8/1895	0.023
	167021	PLC-gamma1 signalling	5/229	8/1895	0.023
	212718	EGFR interacts with phospholipase C-gamma	5/229	8/1895	0.023

	186763	Downstream signal transduction	13/229	44/1895	0.029
	909733	Interferon alpha/beta signaling	8/229	20/1895	0.029
	1251932	PLCG1 events in ERBB2 signaling	5/229	9/1895	0.037
	5654219	Phospholipase C-mediated cascade	5/229	9/1895	0.037
	5654221	Phospholipase C-mediated cascade; FGFR2	5/229	9/1895	0.037
	5654227	Phospholipase C-mediated cascade; FGFR3	5/229	9/1895	0.037
	5654228	Phospholipase C-mediated cascade; FGFR4	5/229	9/1895	0.037
	140877	Formation of Fibrin Clot (Clotting Cascade)	4/229	6/1895	0.044
	392499	Metabolism of proteins	45/229	253/1895	0.047
IC6	198933	Immunoregulatory interactions between a Lymphoid and a non-Lymphoid cell	12/171	34/1895	0.010
	72766	Translation	18/171	87/1895	0.046
	156827	L13a-mediated translational silencing of Ceruloplasmin expression	15/171	66/1895	0.046
	157279	3' -UTR-mediated translational regulation	15/171	66/1895	0.046
	113510	E2F mediated regulation of DNA replication	5/171	9/1895	0.046
	1640170	Cell Cycle	29/171	175/1895	0.046
	72706	GTP hydrolysis and joining of the 60S ribosomal subunit	15/171	67/1895	0.046
	69298	Association of licensing factors with the pre-replicative complex	4/171	6/1895	0.046
	72613	Eukaryotic Translation Initiation	15/171	69/1895	0.046
	72737	Cap-dependent Translation Initiation	15/171	69/1895	0.046
