# Supplementary material for: Affected pathways and transcriptional regulators in gene expression response to an ultra-marathon trail: Global and independent activity approaches
Source: PLoS One. 2017 Oct 13;12(10):e0180322. doi: 10.1371/journal.pone.0180322 (PMC5640184; doi:10.1371/journal.pone.0180322)
Supplement: S11 Table — (PDF) [file pone.0180322.s017.pdf]

S11 Table. List of the statistically overrepresented transcriptional regulators (TRs) obtained per IC after ICA. Three ICs (IC1, IC3 and IC4) among the computed six components shown enriched TRs. TR symbol is indicated for each TR in the list. *Gene Ratio* indicates the number of target genes regulated by the TR within the specific list of 509 major contributor genes which appear in customized TR database obtained from ORA per IC. *Bg Ratio* refers to the number of target genes regulated by the TR within the background (4,772 genes included in the customized database for TREA among the 5,084 differential genes). TRs are sorted based on the adj p-value obtained (FDR).

#IC	TR symbol	GeneRatio	BgRatio	p.adj	p.adj limma
IC1	TAL1	128/450	864/4772	<0.001	Not ranked by limma
	ESR1	201/450	1656/4772	<0.001	Not ranked by limma
	SPI1	149/450	1190/4772	<0.001	<0.001
	CTCF	383/450	3720/4772	<0.001	Not ranked by limma
	GATA2	182/450	1547/4772	0.001	Not ranked by limma
	FOXA1	288/450	2685/4772	0.002	Not ranked by limma
	GATA3	219/450	1981/4772	0.005	0.018
	SMARCA4	388/450	3859/4772	0.006	Not ranked by limma
	STAT1	310/450	2972/4772	0.006	0.005
	TP53	68/450	548/4772	0.036	Not ranked by limma
	TRIM28	91/450	772/4772	0.038	0.001
IC3	GATA2	186/470	1547/4772	0.017	Not ranked by limma
IC4	SMARCA4	412/468	3859/4772	<0.001	Not ranked by limma
	ETS1	365/468	3453/4772	0.044	0.007
