# Supplementary material for: Affected pathways and transcriptional regulators in gene expression response to an ultra-marathon trail: Global and independent activity approaches
Source: PLoS One. 2017 Oct 13;12(10):e0180322. doi: 10.1371/journal.pone.0180322 (PMC5640184; doi:10.1371/journal.pone.0180322)
Supplement: S12 Table — (PDF) [file pone.0180322.s018.pdf]

S12 Table. List of the statistically overrepresented Reactome pathways obtained for IC2 and IC5 after removing first line of variance. ID and description pathway is enclosed in the table. *Gene Ratio* indicates the number of genes annotated to a pathway within the specific list of differential genes among the 509 major contributors that are included in the database (i.e. 190 for IC2). *Bg Ratio* refers to the number of genes annotated to a pathway within the background (all differential genes included in the database which is a total of 1895 elements among 5084).

#IC	ID	Description	Gene Ratio	BgRatio	p.adj
IC2	74160	Gene Expression	61/190	359/1895	0.002
	72766	Translation	22/190	87/1895	0.006
	72689	Formation of a pool of free 40S subunits	17/190	61/1895	0.010
	156902	Peptide chain elongation	15/190	53/1895	0.011
	156842	Eukaryotic Translation Elongation	15/190	54/1895	0.011
	156827	L13a-mediated translational silencing of Ceruloplasmin expression	17/190	66/1895	0.011
	157279	3' -UTR-mediated translational regulation	17/190	66/1895	0.011
	168254	Influenza Infection	17/190	66/1895	0.011
	72706	GTP hydrolysis and joining of the 60S ribosomal subunit	17/190	67/1895	0.012
	72613	Eukaryotic Translation Initiation	17/190	69/1895	0.013
	72737	Cap-dependent Translation Initiation	17/190	69/1895	0.013
	5663205	Infectious disease	30/190	159/1895	0.013
	392499	Metabolism of proteins	42/190	253/1895	0.013
	168255	Influenza Life Cycle	16/190	65/1895	0.017
	1799339	SRP-dependent cotranslational protein targeting to membrane	16/190	66/1895	0.019
	1428517	The citric acid (TCA) cycle and respiratory electron transport	13/190	49/1895	0.024
	168273	Influenza Viral RNA Transcription and Replication	15/190	63/1895	0.029
	163200	Respiratory electron transport, ATP synthesis by chemiosmotic coupling, and heat production by uncoupling proteins.	10/190	33/1895	0.029
	192823	Viral mRNA Translation	13/190	51/1895	0.029
	72764	Eukaryotic Translation Termination	13/190	51/1895	0.029
	927802	Nonsense-Mediated Decay (NMD)	14/190	58/1895	0.030
	975957	Nonsense Mediated Decay (NMD) enhanced by the Exon Junction Complex (EJC)	14/190	58/1895	0.030
	5368286	Mitochondrial translation initiation	11/190	40/1895	0.032
	975956	Nonsense Mediated Decay (NMD) independent of the Exon Junction Complex (EJC)	13/190	53/1895	0.033
	389957	Prefoldin mediated transfer of substrate to CCT/TriC	5/190	10/1895	0.033
	389958	Cooperation of Prefoldin and TriC/CCT in actin and tubulin folding	5/190	10/1895	0.033
	390466	Chaperonin-mediated protein folding	5/190	10/1895	0.033
	74159	Transcription	15/190	68/1895	0.043
	73857	RNA Polymerase II Transcription	12/190	49/1895	0.045
	5368287	Mitochondrial translation	11/190	43/1895	0.046
	391251	Protein folding	5/190	11/1895	0.048
IC5	975956	Nonsense Mediated Decay (NMD) independent of the Exon Junction Complex (EJC)	15/190	53/1895	0.025
	5334118	DNA methylation	5/190	7/1895	0.025
	5625886	Activated PKN1 stimulates transcription of AR (androgen receptor) regulated genes KLK2 and KLK3	5/190	7/1895	0.025
	5625740	RHO GTPases activate PKNs	7/190	15/1895	0.025
	192823	Viral mRNA Translation	14/190	51/1895	0.025
	72764	Eukaryotic Translation Termination	14/190	51/1895	0.025
	927802	Nonsense-Mediated Decay (NMD)	15/190	58/1895	0.025
	975957	Nonsense Mediated Decay (NMD) enhanced by the Exon Junction Complex (EJC)	15/190	58/1895	0.025
	156902	Peptide chain elongation	14/190	53/1895	0.028
	156842	Eukaryotic Translation Elongation	14/190	54/1895	0.031
	168273	Influenza Viral RNA Transcription and Replication	15/190	63/1895	0.048
